# Downregulation of Mfn2 Contributes to Chronic Postsurgical Pain via Inducing the Pyroptosis of GABAergic Neurons in the Spinal Cord

**DOI:** 10.1111/cns.70508

**Published:** 2025-07-09

**Authors:** Yingjie Hu, Xiao He, Hu Zang, Yuye Chen, Li Li, Tongtong Liu, Li Wan, Chang Zhu, Wenlong Yao

**Affiliations:** ^1^ Department of Anesthesiology and Pain Medicine, Hubei Key Laboratory of Geriatric Anesthesia and Perioperative Brain Health, and Wuhan Clinical Research Center for Geriatric Anesthesia Tongji Hospital, Tongji Medical College, Huazhong University of Science and Technology Wuhan China; ^2^ Department of Physiology Hubei University of Chinese Medicine Wuhan China

**Keywords:** chronic postsurgical pain, GABAergic neuron, Mfn2, mitochondrial dysfunction, pyroptosis

## Abstract

**Background:**

Chronic postoperative pain (CPSP) is a significant public health issue due to the complex pathophysiological mechanism. Existing evidence has pointed out that the loss of gamma‐aminobutyric acid‐ergic (GABAergic) neurons played a critical role in various neuropathic pain models. Previous studies also found that pyroptosis‐mediated neuroinflammation was involved in neuropathological pain. However, it remains unclear what the relationship is between pyroptosis and the loss of spinal GABAergic neurons in CPSP. This study aimed to investigate the role and mechanism of GABAergic neuron pyroptosis in CPSP.

**Methods:**

We used skin/muscle incision and retraction (SMIR) to establish the CPSP model in rats. Mechanical allodynia was assessed using the Von Frey test. Western blotting, quantitative real‐time polymerase chain reaction (qRT‐PCR), immunofluorescence, biochemical assay, and transmission electron microscope (TEM) were employed to investigate the role and mechanism of GABAergic neuron pyroptosis during CPSP.

**Results:**

We observed the pyroptosis of GABAergic neurons in the spinal cord following SMIR. Intrathecal administration of the GSDMD inhibitor decreased the pyroptosis of GABAergic neurons in the spinal cord and reversed SMIR‐induced mechanical allodynia. In addition, we found that SMIR induced a significant decrease in the level of Mfn2 in the neurons, accompanied by mitochondrial dysfunction and reactive oxygen species (ROS) accumulation in SMIR rats. Intrathecal injection of the Mfn2 activator reduced mitochondrial dysfunction and ROS, alleviated the pyroptosis of GABAergic neurons in the spinal cord, which alleviated the SMIR‐induced mechanical allodynia.

**Conclusions:**

Our study demonstrated that downregulation of Mfn2 leads to mitochondrial dysfunction and ROS accumulation, which promotes the pyroptosis of spinal GABAergic neurons and the development of chronic pain.

## Graphical abstract

Schematic diagram of the role and activation mechanism of GABAergic neuron pyroptosis in CPSP. Downregulation of Mfn2 induces the GABAergic neuron pyroptosis which is involved in the development of chronic postoperative pain by promoting mitochondrial dysfunction and ROS accumulation.

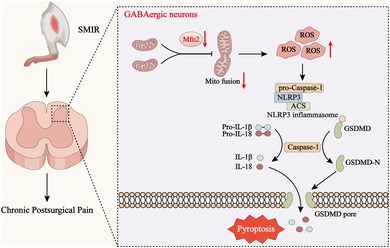

**Abbreviations**
ANOVA: analysis of variance
ATP: adenosine triphosphate
CPSP: chronic postoperative pain
DSF: disulfiram
Drp1: dynamin‐related protein 1
GABA: gamma‐aminobutyric acid
GSDMD: gasdermin D
GAPDH: glyceraldehyde‐3‐phosphate dehydrogenase
GFAP: glial fibrillary acidic protein
GAD1: glutamic acid decarboxylase 1
Iba1: ionized calcium‐binding adapter molecule 1
i.t.: intrathecal injection
IF: immunofluorescence
IL‐1β: interleukin‐1β
IL‐18: interleukin‐18
MMP: mitochondrial membrane potential
mtDNA: mitochondrial DNA
Mfn2: mitofusin‐2
NLRP3: nod‐like receptor pyrin domain‐containing
NeuN: neuronal nuclear protein
PWT: paw withdrawal threshold
PBS: phosphate buffered saline
qPCR: quantitative polymerase chain reaction
ROS: reactive oxygen species
SMIR: skin/muscle incision and retraction
VGLUT2: vesicular glutamate transporter 2
WB: western blot

## 1 Introduction

Chronic postsurgical pain (CPSP) is characterized by a persistent pain occurring at or around the surgical site for at least 3 months postsurgery, excluding other potential causes, such as chronic infection, recurrence of malignancy, and so on [1]. Statistics show that approximately 10% of surgical patients suffer from moderate–to‐severe CPSP [2]. For certain types of surgery, such as amputations, thoracotomies, and mastectomies, the incidence can be higher, ranging from 30% to 85% [1]. CPSP complicates recovery and impairs the function and quality of life of the affected [3]. However, current strategies to treat and prevent CPSP are far from satisfactory [4, 5]. Therefore, understanding the pathological mechanisms underlying CPSP is crucial for developing effective therapeutic interventions.

It has been known that γ‐aminobutyric acid (GABA)‐ergic neurons are important in the development and maintenance of chronic pain [6, 7]. Specifically, the reduction in the inhibitory neurotransmitter GABA and the loss of GABAergic neurons propose an imbalance of excitatory and inhibitory neurotransmission, which is associated with hyperalgesia and allodynia [8, 9]. Recently, pyroptosis, a mode of programmed cell death closely associated with inflammation, plays a key role in neuropathic and inflammatory pain [10, 11]. Gasdermin D (GSDMD) is a key executioner of the classical pyroptosis pathway, and its activation depends on the assembly of the NOD‐like receptor pyrin domain containing 3 (NLRP3) inflammasome and the activation of Caspase‐1. The N‐terminal fragment of GSDMD (GSDMD‐N) specifically targets the cell membrane, leading to membrane disruption and the release of pro‐inflammatory factors [12, 13]. However, it remains unclear whether the loss of spinal GABAergic neurons is attributed to pyroptosis in CPSP.

Mitofusin‐2 (Mfn2) is an essential fusion protein located on the outer mitochondrial membrane, playing a critical role in regulating mitochondrial morphology, function, and biological processes [14]. Previous studies have demonstrated that Mfn2 dysfunction contributes to the progression of various neurodegenerative diseases [15, 16]. A recent study has shown that a reduction in Mfn2 expression impairs mitochondrial function, leading to the activation of the NLRP3 inflammasome and initiating the innate immune response, particularly in conditions such as myocardial ischemia–reperfusion injury [17]. However, the roles and molecular mechanisms of Mfn2 in chronic pain remain largely unexplored.

Therefore, we investigated the role of spinal GABAergic neuron pyroptosis in the development of CPSP and the underlying activation mechanisms. In the present study, we established a CPSP model in rats using skin/muscle incision and retraction (SMIR), and examined the changes in the pyroptosis‐related proteins, the number of GABAergic neurons, mitochondrial function, and Mfn2 in the spinal cord. To assess the effect of inhibiting GABAergic neuron pyroptosis on CPSP, we administered disulfiram (DSF), a gasdermin D (GSDMD) inhibitor. Finally, the Mfn2 activator was used to explore the effect of Mfn2 upregulation on mitochondrial dysfunction and pyroptosis of GABAergic neurons during CPSP.

## 2 Materials and Methods

### 2.1 Animals

Male Sprague–Dawley rats weighing 200–220 g from Tongji Medical College, Huazhong University of Science and Technology (Wuhan, China) were used in this study. The rats were maintained under standard conditions, including a 12‐h light/dark cycle, 50% relative humidity, and a controlled temperature of 22°C–25°C, with unrestricted access to food and water. All experimental procedures complied with the National Institutes of Health Guidelines for the Care and Use of Laboratory Animals and were approved by the Experimental Animal Care and Use Committee of Tongji Medical College, Huazhong University of Science and Technology.

### 2.2 Skin/Muscle Incision and Retraction (SMIR)

According to Flatters' method [18], we carried out the skin/muscle incision and retraction (SMIR) surgery. Briefly, rats were first anesthetized by 1% sodium pentobarbital (50 mg/kg, i.p.), and the hair on the right inner thigh was removed. Then, we found the saphenous vein in the inner thigh of the rat, and a 1.5–2 cm incision was made about 4 mm into the skin to expose the thigh muscle. The superficial muscles (gracilis) were subsequently divided 7–10 mm using blunt dissection tools to expose the deep muscle (adductor) fascia. Muscle and nodal tissues were further bluntly separated, and a microanatomical retractor (Biomedical Research Instruments Inc., USA). After ensuring that all tips of the microanatomical retractor were placed under the superficial muscles, the thigh skin and superficial muscles were contracted by 2 cm to expose the superficial submuscular adductor fascia for 1 h. Rats were covered with large absorbent tape pads during exposure to reduce water evaporation from the surgical site and heat loss in rats. After 1 h, muscles and skin were sutured with Vicryl sutures of 3.0 and 4.0, respectively. In the Sham group, only skin and superficial muscles were incised without traction.

### 2.3 Paw Withdrawal Threshold Test (PWT)

As previously described, the mechanical paw withdrawal threshold (PWT) test was utilized to assess mechanical allodynia was assessed using Chaplan et al. [19] Specifically, rats were placed in a separate opaque box with a metal grid at the bottom, ensuring that the rats' hind paw could fully reach the metal grid. After the rats were habituated to such an environment for 30 min, different von Frey filaments (4–15 g) were slowly applied to the mid‐plantar region of the right paw from the grid for about 3 s. Positive findings included sudden paw retraction, shivering, and licking. At least five more tests are required after a positive result. Finally, 50% PWT was determined by the up‐down method [19].

### 2.4 Intrathecal Catheterization and Drug Administration

Intrathecal catheterization was performed 5 days before SMIR, as previously described [20]. Specifically, after being fully anesthetized (1% sodium pentobarbital, 50 mg/kg, i.p.), the skin and muscle between the L4–L5 spinous processes were then cut under sterile conditions to fully expose the disc space, and a sterile polyethylene catheter (PE‐10; outer diameter, 0.5 mm, inner diameter, 0.3 mm; Anilab Software & Instruments, Ningbo, China) was inserted into the subarachnoid space. Markers of success with the intrathecal catheterization included tail flick reflex, cerebrospinal fluid outflow, and temporary paralysis of both hind limbs caused by 2% lidocaine (10 μL). Finally, the catheter is fixed, and the other end of the catheter is fixed subcutaneously to facilitate drug delivery. Any animals with unsuccessful catheterization and nerve injury were excluded.

Drug preparation used in this study is shown below. Disulfiram (DSF, HY‐B0240, MedChemExpress, China), a specific inhibitor of GSDMD, was dissolved in 10% dimethyl sulfoxide (DMSO) in sterile saline. MASM7 (HY‐W187305, MedChemExpress, China), a mitofusin activator, was dissolved in 5% DMSO and 5% Tween 80 in sterile saline. In this study, we tend to explore the spinal cord level mechanisms. Therefore, we chose intrathecal administration rather than intraperitoneal or other modalities. Based on previous relevant drug studies [21, 22], different doses of DSF (10, 50, and 100 μg/10 μL, intrathecal) and MASM7 (2.5, 10, and 25 μg/10 μL, intrathecal) were administered as a single treatment on day 1 after SMIR. Then, 5 μg of DSF and 10 μg of MASM7 were administered daily for continuous treatment on days 1–7 after SMIR. Intrathecal injection of the corresponding vehicle was used as the carrier group.

### 2.5 Western Blot

After deep anesthesia and sacrifice, the L3–L5 spinal segments were swiftly excised and transferred to radioimmunoprecipitation assay (RIPA) lysis buffer (Boster Biological Technology, Wuhan) containing protease and phosphatase inhibitors for homogenization. Next, after centrifuging at 12 000 ***g*** and 4°C for 15 min, the supernatant was collected. Then the protein concentration was detected using the BCA protein assay kit (Boster, Wuhan, China). All protein samples were boiled in a 90°C water bath for 10 min after adding SDS loading buffer (EpiZyme, Shanghai, China) and stored at −80°C. Equal amounts of protein (30 μg) were loaded and resolved on 8%–12% SDS‐PAGE gel electrophoresis and subsequently transferred into polyvinylidene fluoride membranes (Millipore, Billerica, MA, USA). Then, nonspecific protein binding sites were blocked with 5% skim milk or BSA in 0.1% Tris‐buffered saline and Tween 20 at room temperature and subsequently incubated with specific primary antibodies overnight at 4°C on a shaker. Antibodies used in this study are shown in Table 1.

**TABLE 1:** The detailed information on antibodies.

Antibody	Source	Host	Identifier	Dilution	Applications
NLRP3	Wanleibio	Rabbit	WL02635	1:1000	WB
Caspase1 + P20 + P10	Santa Cruz	Mouse	sc‐56036	1:200	WB
GSDMD (full‐length + *N* terminal)	Signalway Antibody	Rabbit	55651	1:1000	WB
GSDMD (full‐length + *N* terminal)	Abclonal Technology	Rabbit	A24476	1:50	IF
GAD1	Proteintech	Mouse	67648‐1‐Ig	1:5000 1:400	WB IF
VGLUT2	Novus Biologicals	Rabbit	NBP3‐13164	1:1000 1:300	WB IF
Mfn1	Proteintech	Rabbit	13798‐1‐AP	1:2000	WB
Mfn2	Abclonal Technology	Rabbit	A19678	1:2000	WB
Mfn2	Proteintech	Rabbit	11186‐1‐AP	1:50	IF
DRP1 (phosphor‐ser637)	Signalway Antibody	Rabbit	11842	1:1000	WB
DRP1	Abclonal Technology	Rabbit	A21968	1:2000	WB
GAPDH	Proteintech	Mouse	60004‐1‐Ig	1:10000	WB
Iba1	Abcam	Goat	ab5076	1:100	IF
NeuN	Abcam	Mouse	ab104224	1:50	IF
GFAP	Cell Signaling Technology	Mouse	3670	1:100	IF
Anti‐ mouse IgG HRP	Proteintech	Goat	SA00001‐1	1:10000	WB
Anti‐rabbit IgG HRP	Proteintech	Goat	SA00001‐2	1:10000	WB
Alexa Fluor 488‐labeled anti‐mouse IgG	Jackson ImmunoResearch	Goat	AB_2338840	1:300	IF
Alexa Fluor 488‐labeled anti‐mouse IgG	Jackson ImmunoResearch	Donkey	AB_2341099	1:100	IF
Alexa Fluor 488‐labeled anti‐rabbit IgG	Jackson ImmunoResearch	Donkey	AB_2338046	1:200	IF
Alexa Fluor 594‐labeled anti‐rabbit IgG	Jackson ImmunoResearch	Goat	AB_2338059	1:400	IF
Alexa Fluor 594‐labeled anti‐rabbit IgG	Jackson ImmunoResearch	Donkey	AB_2340621	1:100	IF
Alexa Fluor Cy3‐labeled anti‐mouse IgG	Jackson ImmunoResearch	Goat	AB_2338680	1:200	IF

The following day, after the membranes were washed in Tris‐buffered saline with Tween 20 (TBST, 0.1%) for three times, they were incubated with the corresponding HRP goat anti‐rabbit IgG antibody or HRP goat anti‐mouse IgG antibody for 2 h at room temperature. Finally, positive signals were detected using the SuperLumia ECL Plus HRP Substrate Kit (Abbkine, Wuhan, China) and ChemiDoc XRS + imaging system (Bio‐Rad, USA). For some proteins with similar target molecular weights and GAPDH as an internal control, antibody incubation was repeated after Western blot fast stripping buffer (EpiZyme, Shanghai, China). All band intensities were analyzed using software, and target protein responses were quantified and normalized with GAPDH. Meanwhile, the protein expression of Sham and Sham + Vehicle was set to 1.

### 2.6 Immunofluorescence

Under deep anesthesia, the rat hearts were perfused with 0.1 M phosphate buffered saline (PBS) and 4% paraformaldehyde (PFA), respectively. Then, L3–L5 spinal segments were fixed in 4% PFA for 24 h at 4°C, followed by cryoprotection in 30% sucrose until tissues sank. Next, samples were embedded in OCT compound and sectioned at 20 μm thickness using a cryostat (CM1900, Leica, Wetzlar, Germany). Sections were permeabilized with 0.1% Triton X‐100 in PBS for 15 min and blocked nonspecific binding sites of proteins with 5% goat or donkey serum for 1 h at room temperature. Then, tissue sections were incubated with primary antibodies mixture overnight at 4°C. After three washes with PBS, sections were incubated with Alexa Fluor‐conjugated secondary antibodies for 2 h at room temperature in the dark. Nuclei were counterstained with DAPI for 5 min. Following additional PBS washes, slides were mounted using ProLong Gold antifade reagent. Images were captured using a confocal microscope (BX51, OLYMPUS, Japan) with the same condition. Four nonadjacent sections (three rats per group) were randomly selected from each animal and images were processed with Adobe Photoshop. Immunofluorescence intensity and the number of positive cell staining were calculated by ImageJ (National Institutes of Health, MD, USA). The antibodies used are listed in Table 1.

### 2.7 Quantitative Real‐Time PCR (q‐PCR)

Total RNA was isolated from the L3–L5 spinal segments using TRIzol reagent (Takara, Shiga, Japan) following the manufacturer's protocol. RNA concentration and purity were assessed using a spectrophotometer (NanoDrop 2000; Thermo Scientific, Waltham, MA, USA). One microgram of total RNA was used to synthesize cDNA using the First‐Strand cDNA Synthesis Kit (Vazyme Biotech, Nanjing, China). Quantitative real‐time PCR was performed using the SYBR qPCR Master Mix (Abclonal Technology) following the manufacturer's instructions. Gene expression levels were normalized to GAPDH as the endogenous control and analyzed using the 2^−ΔΔCt^ method. Primer sequences are listed in Table 2.

**TABLE 2:** The primer sequences used for quantitative PCR assay.

Gene	Forward primer sequence (5′ to 3′)	Reverse primer sequence (5′ to 3′)
Il‐1β	TCAGGCAGGCAGTATCACTC	CATGAGTCACAGAGGATGGG
Il‐18	AACAGCCAACGAATCCCAGAC	TTGTTTTTACAGGAGAGGGTAGACA
GAPDH	TGGCCTTCCGTGTTCCTACC	CGCCTGCTTCACCACCTTCT
NADH	GAGCCGTTGCCCAAACCATCTC	AGAAGGAGCCGCTTATTAGGAGGAC
β‐globin	GCAGGTTGGTATCCAGGTTACAAGG	AGCAGCCTACAAAGGGAAACATAGC

### 2.8 Mitochondrial Isolation

Mitochondria were isolated from L3 to L5 spinal segments of rats using a mitochondrial isolation kit (Beyotime, Shanghai, China) according to the manufacturer's protocol. Briefly, tissues were homogenized in ice‐cold isolation buffer using a glass Dounce homogenizer. The homogenate was centrifuged at 1000 ***g*** for 10 min at 4°C to remove nuclei and debris. The supernatant was further centrifuged at 12 000 ***g*** for 15 min at 4°C to pellet the mitochondria. The mitochondrial pellet was washed with isolation buffer and recentrifuged at 12 000 ***g*** for 10 min. The final mitochondrial pellet was resuspended in an appropriate volume of storage buffer, and protein concentration was determined using the BCA protein assay kit (Boster, Wuhan, China). Isolated mitochondria were stored on ice and used immediately for subsequent experiments or stored at −80°C for later use.

### 2.9 Cytosolic DNA Isolation and DNA Copy Number Analysis

According to the above method, during the isolation of mitochondria, the supernatant after the second centrifugation was collected and centrifuged twice at 12 000 ***g***, 4°C, and the supernatant was the cytosolic component. Then, cytosolic DNA from the spinal cord was extracted with the DNeasy Blood and Tissue Kit (Qiagen) according to the manufacturer's instructions. Next, based on a previously described method, nuclei DNA and mtDNA were detected using SYBR qPCR Master Mix by different primer sequence designs (Table 2) [23].

### 2.10 Mitochondrial Membrane Potential (MMP)

Mitochondrial membrane potential changes were detected by a mitochondrial membrane potential assay kit (Beyotime, Shanghai, China). Following mixing purified mitochondria with JC‐1 staining working solution according to the manufacturer's protocol, fluorescence intensities of JC‐1 monomers (*λ*_ex_ 490 nm and *λ*_em_ 530 nm) and aggregates (*λ*_ex_ 525 nm and *λ*_em_ 590 nm) were detected using a multifunctional enzyme labeler (Synergy2, Biotek, USA), respectively. The ratio of red (aggregate)/green (monomer) fluorescence represents the level of MMP, and the fluorescence intensity of sham or SMIR + Vehicle is set to 1.

### 2.11 Detection of MitoSox Red in the Spinal Cord Dorsal Horn

Mitochondrial ROS production was measured using MitoSox Red (Invitrogen, Shanghai, China) according to previous research methods [24]. Specifically, MitoSox Red was diluted in 2% dimethylsulfoxide (DMSO) in 33 μM saline. Rats were injected intrathecally with 30 μL MitoSox Red into the L3–L5 segment, and 70 min later, the rats were perfused with cold PBS followed by 4% ice‐cold PFA. Next, the spinal cords were fixed and dehydrated in the dark and finally cut transversely to a thickness of 20 μm, referring to the immunofluorescence method described above. Sections were imaged under the same conditions with a fluorescence microscope (DP70, Olympus, Japan). The area of positive cells in the spinal dorsal horn was calculated by Image J (National Institutes of Health, MD, USA).

### 2.12 Transmission Electron Microscopy (TEM)

Rats were euthanized after deep anesthesia, and the spinal cords were quickly removed and sliced into 1–2 mm sections. Then the slices were fixed in 2.5% glutaraldehyde for 4 h and underwent secondary fixation in 1% osmium tetroxide for 1 h. Next, the samples underwent gradient dehydration with ethanol (30%, 50%, 70%, 90%, and 100%) for 15 min each, and then were treated with xylene three times. Subsequently, samples were embedded in epoxy resin and cured for 24 h. Ultra‐thin sections (50–100 nm) were prepared using an ultramicrotome and placed on electron microscope grids. Observations were conducted using a transmission electron microscope (Thermo, USA) at an operating voltage of 80 kV, documenting cellular structures and their interrelations.

### 2.13 Experimental Designs and Animal Groups

**FIGURE 1:**
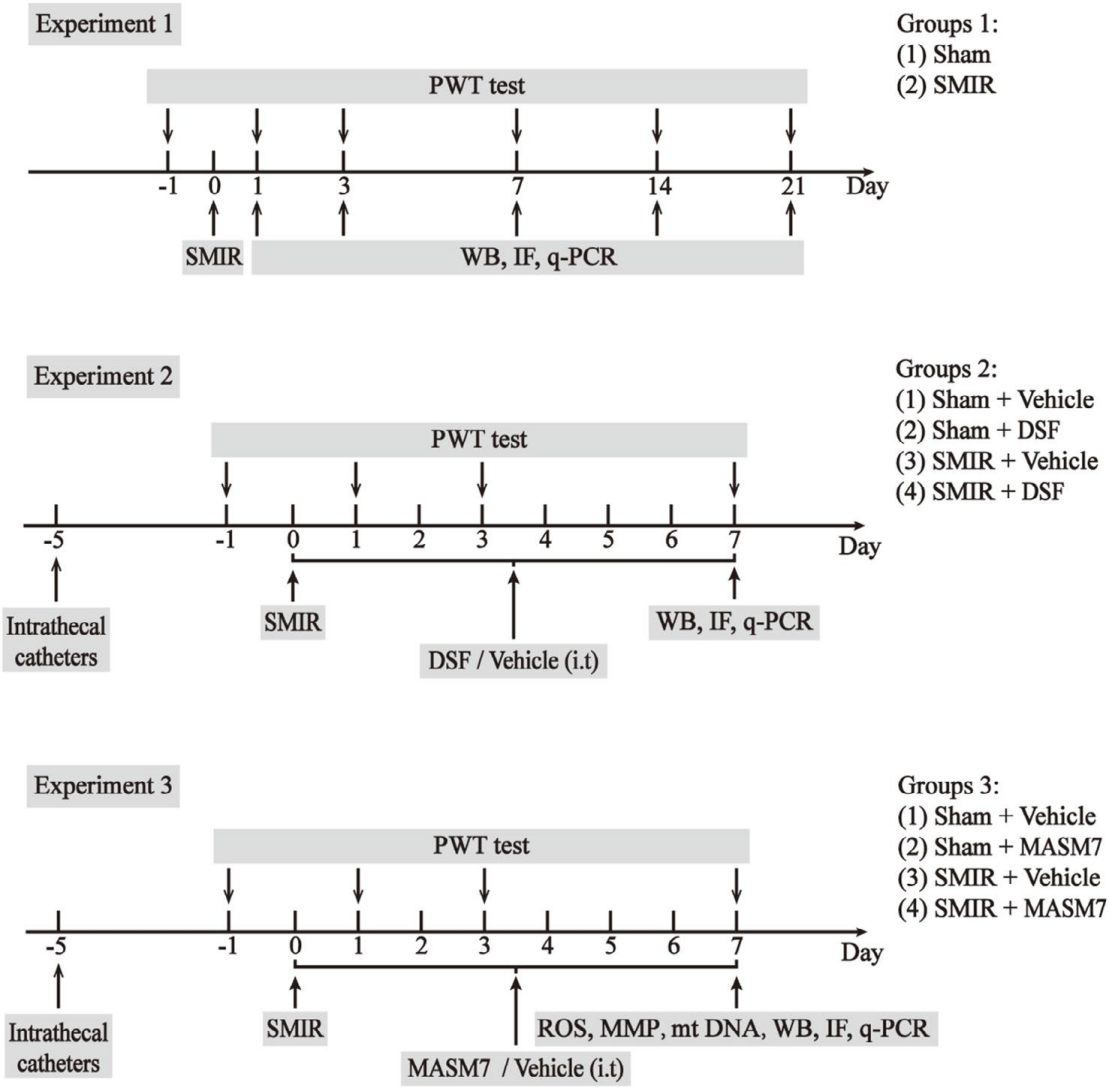
Experimental designs and animal groups. Experiment 1: The changes of the NLRP3/Caspase‐1/GSDMD pathway, GABAergic neurons, and mitochondrial dynamics‐related proteins in the spinal cord of rats with SMIR. Experiment 2: The effects of GSDMD inhibitor (DSF) on mechanical allodynia and pyroptosis of GABAergic neurons in rats with SMIR. Experiment 3: The effects of Mfn2 activator (MASM7) on mechanical allodynia, mitochondrial function, and the pyroptosis of GABAergic neurons in rats with SMIR. DSF, disulfiram; i.t., intrathecal injection; IF, immunofluorescence; MMP, mitochondrial membrane potential; mtDNA, mitochondrial DNA; PWT, paw withdrawal threshold; qPCR, quantitative polymerase chain reaction; ROS, reactive oxygen species; SMIR, skin/muscle incision and retraction; WB, western blot.

The experimental design and animal groups are presented in Figure 1.

#### 2.13.1 Experiment 1: The Changes of the NLRP3/Caspase‐1/GSDMD Pathway, GABAergic Interneurons, and Mitochondrial Dynamics‐Related Proteins in the Spinal Cord of Rats With SMIR

Fifty‐four rats were randomly divided into Sham or SMIR groups. Paw withdrawal thresholds (PWTs) were measured at days 1, 3, 7, 14, and 21 after SMIR. Then extracting the L4–L6 segments from the sham and SMIR groups at the corresponding time points for Western blot and qPCR. The Sham and SMIR rats at day 7 were performed for immunofluorescence colocalization.

#### 2.13.2 Experiment 2: The Effects of GSDMD Inhibitor (DSF) on Mechanical Allodynia and the Pyroptosis of GABAergic Interneurons in Rats With SMIR

To determine an effective dose of DSF for relieving CPSP, 24 rats were randomly categorized into four groups (*n* = 6 per group): SMIR + Vehicle, SMIR + 10 μg DSF, SMIR + 50 μg DSF, and SMIR + 100 μg DSF. Vehicle or different concentrations of DSF were administered intrathecally as a single dose on day 3 after SMIR, and PWT was detected within 8 h after administration. Subsequently, to determine whether repeated injection of DSF (50 μg) could reverse mechanical allodynia in SMIR rats, 48 rats were randomly divided into four groups (*n*=12 per group): Sham + Vehicle; Sham + DSF; SMIR + Vehicle; SMIR + DSF. DSF (50 μg) was administered once daily for seven consecutive days after surgery, with behavioral tests conducted 2 h after injection. Then the spinal cord of the rats was isolated to further biochemical test.

#### 2.13.3 Experiment 3: The Effects of Mfn2 Activator (MASM7) on Mechanical Allodynia, Mitochondrial Function, and the Pyroptosis of GABAergic Interneurons in Rats With SMIR

To explore an effective dose of MASM7 for relieving CPSP, 24 rats were randomly categorized into four groups (*n* = 6 per group): SMIR + Vehicle, SMIR + 2.5 μg MASM7, SMIR + 10 μg MASM7, and SMIR + 25 μg MASM7. Then, 48 rats were randomly divided into four groups (*n* = 12 per group) to investigate the effect of repeated injection of MASM7 (10 μg) on mechanical allodynia, mitochondrial function, and the pyroptosis of GABAergic interneurons in rats with SMIR: Sham + Vehicle, Sham + MASM7, SMIR + Vehicle, SMIR + MASM7. MASM7 (10 μg) was administrated once daily for seven consecutive days after surgery, with behavioral tests conducted 2 h after injection.

### 2.14 Statistical Analyses

All results are expressed as the mean ± standard error of the mean (SEM). Data were analyzed using the IBM SPSS Statistics (version 26.0; IBM Corp., Armonk, NY, USA) and GraphPad Prism version 9 (GraphPad Software, San Diego, CA, USA). To assess the normality of continuous variables, we examined Q‐Q plots and conducted Shapiro–Wilk tests to determine whether the data exhibited significant skewness or kurtosis. Considering the within‐subject correlation inherent in repeated measures, the generalized estimating equation (GEE) models were used to compare changes in PWTs over time between different groups, followed by Bonferroni's post hoc test for group comparisons. Data from immunofluorescence, qPCR, and Western blot analyses were analyzed using one‐way ANOVA with Bonferroni correction for multiple comparisons. For comparisons between two groups, unpaired two‐tailed Student's t‐tests were used. All statistical tests were two‐tailed, and a *p*‐value of < 0.05 was considered statistically significant.

## 3 Results

### 3.1 The GSDMD‐Mediated Pyroptosis Is Activated in the Spinal Cord After SMIR

**FIGURE 2:**
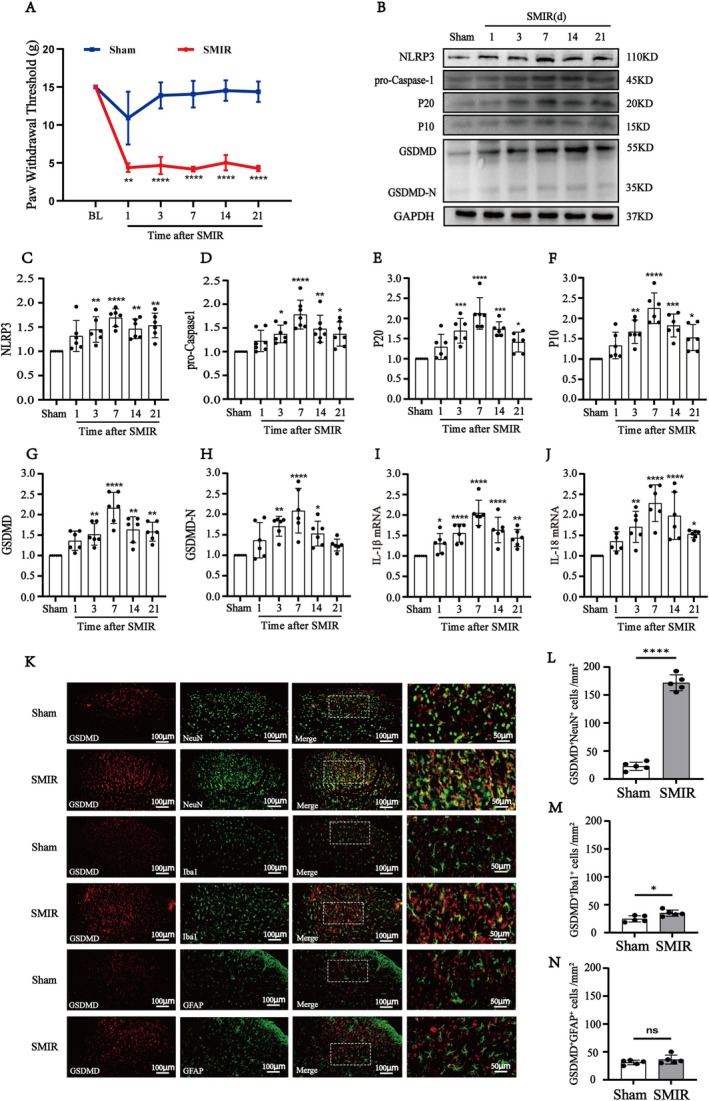
The GSDMD‐mediated pyroptosis is activated in the spinal cord after SMIR. (A) Mechanical allodynia evaluated by PWT was assessed at baseline and 1, 3, 7, 14, and 21 days after surgery (*n* = 8). Generalized estimating equations (GEE) model, followed by Bonferroni's post hoc test, **p < 0.01, ****p < 0.0001 compared with the Sham group. (B) Representative western blot images of the NLRP3‐Caspase1‐GSDMD pathway expression in the sham or SMIR rats. (C, H) Quantification results of western blot demonstrated that the protein levels of NLRP3, caspase‐1, P20, P10, GSDMD, and GSDMD‐N were significantly upregulated from day 3 to day 21 after SMIR, peaking on day 7 (*n* = 6). One‐way ANOVA, followed by Bonferroni's post hoc test, *p < 0.05, **p < 0.01, ***p < 0.001, ****p < 0.0001 compared with the Sham group. (I, J) Quantitative PCR analysis showed the mRNA levels of IL‐1β and IL‐18 in SMIR rats were significantly increased from day 1 to day 21 (*n* = 6). One‐way ANOVA, followed by Bonferroni's post hoc test, *p < 0.05, **p < 0.01, ***p < 0.001, ****p < 0.0001 compared with the Sham group. (K) Double immunofluorescence and statistical analysis of GSDMD with NeuN, Iba1, GFAP in the spinal cord of the sham and SMIR rats. Scale bar: 100 μm and 50 μm. (L–N) Quantification results of immunoreactivity of GSDMD^+^NeuN^+^ and GSDMD^+^Iba1^+^ in the SMIR group were significantly increased compared with the Sham group, while there was no difference in GSDMD^+^GFAP^+^ (*n* = 4). Student's *t*‐test, *p < 0.05, ****p < 0.0001 compared with Sham group. BL, baseline; GFAP, glial fibrillary acidic protein; Iba1, ionized calcium‐binding adapter molecule 1; NeuN, Neuronal nuclei.

Behavioral tests showed no significant differences in the mechanical PWT between the Sham and SMIR groups at baseline. However, the SMIR group exhibited a significant reduction of mechanical PWT from days 3 to day 21 compared with the Sham group (Figure 2A). The findings demonstrated that the SMIR effectively triggered the development of mechanical allodynia.

To investigate the involvement of the GSDMD‐mediated pyroptosis in CPSP, we examined the pyroptosis‐related protein expression levels on days 1, 3, 7, 14, and 21 following SMIR. As shown in Figure 2B–H, Western blot demonstrated that the protein levels of NLRP3, Caspase‐1, and cleaved Caspase‐1 (P10+P20), GSDMD, and GSDMD‐N were significantly upregulated from day 3 to day 21 after SMIR, peaking on day 7. Additionally, compared with the Sham group, the mRNA levels of IL‐1β and IL‐18 were persistently increased from day 1 to day 21 (Figure 2I,J).

Given that GSDMD is a key executor of pyroptosis, we further investigate the cellular localization of GSDMD in the spinal cord. We performed double immunofluorescence of GSDMD with cell‐specific markers, including Iba‐1 for microglia, GFAP for astrocytes, and NeuN for neurons after 7 days of SMIR. As shown in Figure 2K–N, GSDMD was predominantly expressed in neurons, with lower levels observed in microglia and astrocytes. Furthermore, the SMIR group exhibited a significant increase in GSDMD^+^NeuN^+^ immunoreactivity compared with the Sham group. Those results indicated that the pyroptosis of spinal neurons was involved in CPSP development.

### 3.2 Spinal GABAergic Neurons Underwent Pyroptosis After SMIR

**FIGURE 3:**
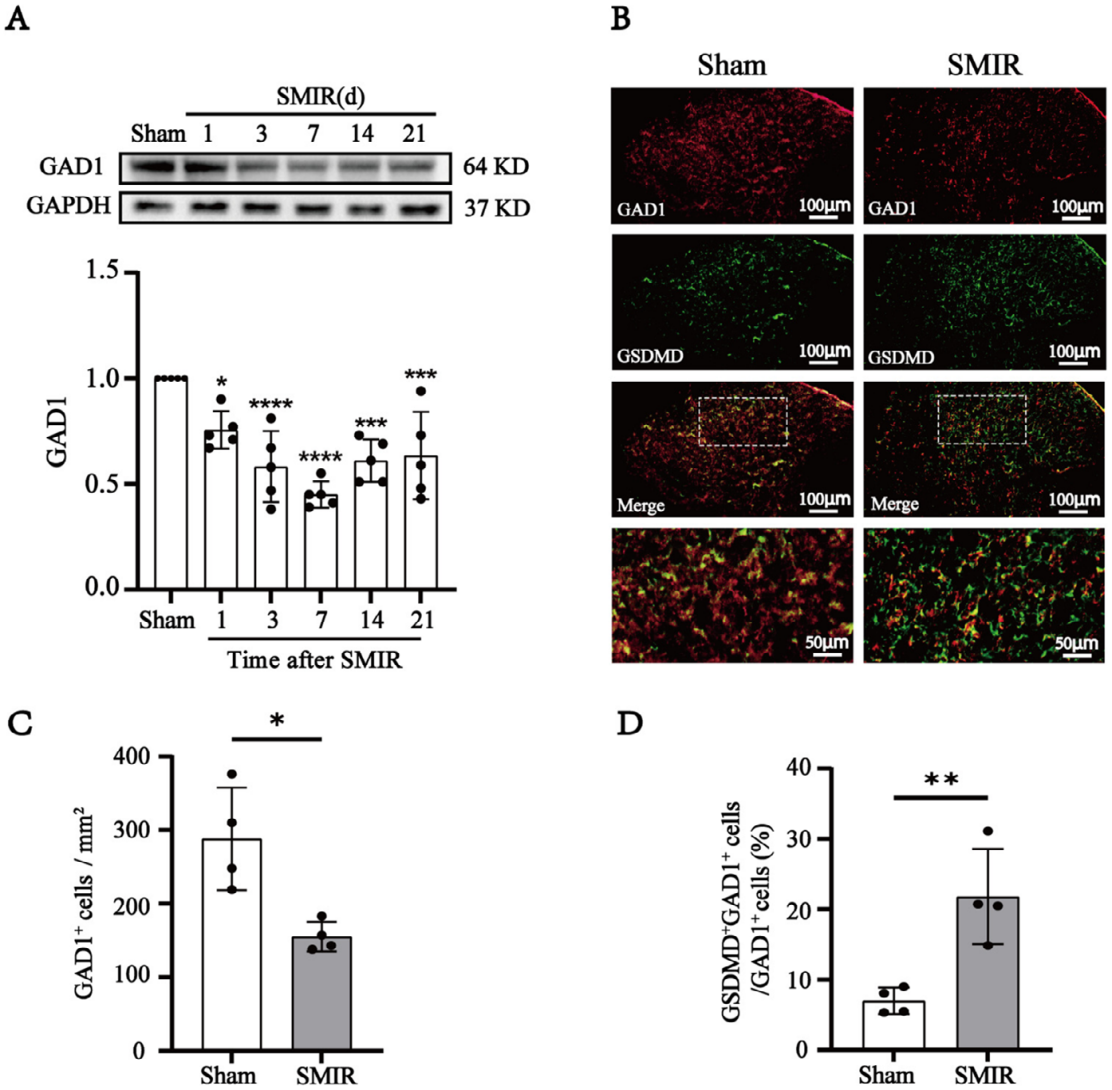
Pyroptosis of GABAergic neurons in the spinal cord of SMIR rats. (A) Western blot showed that the level of GAD1 in the SMIR rats is significantly decreased from days 1 to 21 (*n* = 6). One‐way ANOVA, followed by Bonferroni's post hoc test, *p < 0.05, **p < 0.01, ***p < 0.001, ****p < 0.0001 compared with the Sham group. (B) Double immunofluorescence of GSDMD (green) with GAD1 (red) in the spinal cord of the sham and SMIR rats. Scale bar: 100 μm and 50 μm. (C) The number of GAD1^+^ cells were evidently decreased at lamina III‐IV of the spinal cord dorsal horn in the SMIR rats (*n* = 4). Student's *t*‐test, *p < 0.05 compared with Sham group. (D) There was a higher proportion of GSDMD^+^GAD1^+^ GABAergic neurons in the SMIR group compared to sham rats (*n* = 4). Student's *t*‐test, **p < 0.01 compared with Sham group.

Accumulating evidence indicated that the loss of GABAergic neurons in the spinal cord contributes to the development of persistent hyperalgesia [25, 26]. Therefore, we investigated whether the loss of GABAergic neurons in SMIR rats was associated with pyroptosis. Glutamic acid decarboxylase 1 (GAD1), a GABA‐synthesizing enzyme, is closely linked to both the number and functional changes of GABA neurons [27]. Western blot indicated that compared with the Sham groups, the expression of GAD1 was markedly decreased after SMIR, and lowest at day 7 after SMIR (Figure 3A), while the expression of excitatory neuronal marker vesicular glutamate transporter 2 (VGLUT2) was significantly increased (Figure S2A). To further explore the relationship between pyroptosis and GABAergic neuron loss, we examined the colocalization of GAD1 and GSDMD in the spinal cord (Figure 3B). In line with the results of protein quantitative analysis, the numbers of GAD1^+^ cells were significantly reduced (Figure 3C) and the numbers of VGLUT2^+^ cells were significantly increased (Figure S2B) in the spinal cords of SMIR rats. Notably, a higher proportion of GSDMD^+^GAD1^+^ GABAergic neurons was observed in the SMIR group compared with the Sham group (Figure 3D). These findings indicated that the spinal GABAergic neurons pyroptosis was involved in the SMIR‐induced CPSP.

### 3.3 Intrathecal GSDMD Inhibitor Reversed SMIR‐Induced Mechanical Allodynia via Decreasing the Pyroptosis of Spinal GABAergic Neurons

To test whether activation of the GSDMD‐mediated pyroptosis in spinal cord contributes, at least in part, to the pain behavior of SMIR rats, we intrathecally injected the GSDMD inhibitor disulfiram (DSF) to pharmacologically intervene in the pathway after SMIR. First, DSF (10, 50, 100 μg, intrathecally) or vehicle (10% DMSO) was administered once on day 3 after SMIR to determine the optimal dose. The von Frey test was conducted before DSF injection, and 0.5, 1, 2, 4, 6, and 8 h after injection (Figure S1A). We found that compared with the vehicle group, all three doses of DSF significantly increased ipsilateral PWT in SMIR rats at 1 h after administration. However, the analgesic potency and duration vary among different doses. Moderate (50 μg) and high (100 μg) doses of DSF notably produced a more than 3‐fold increase in PWT, which showed the highest analgesic potency. The analgesic potency peaked at 2 h and persisted for at least 8 h (Figure S1B). Considering the potential cytotoxicity following the cumulative effect of the dose, 50 μg DSF was selected for further investigation.

**FIGURE 4:**
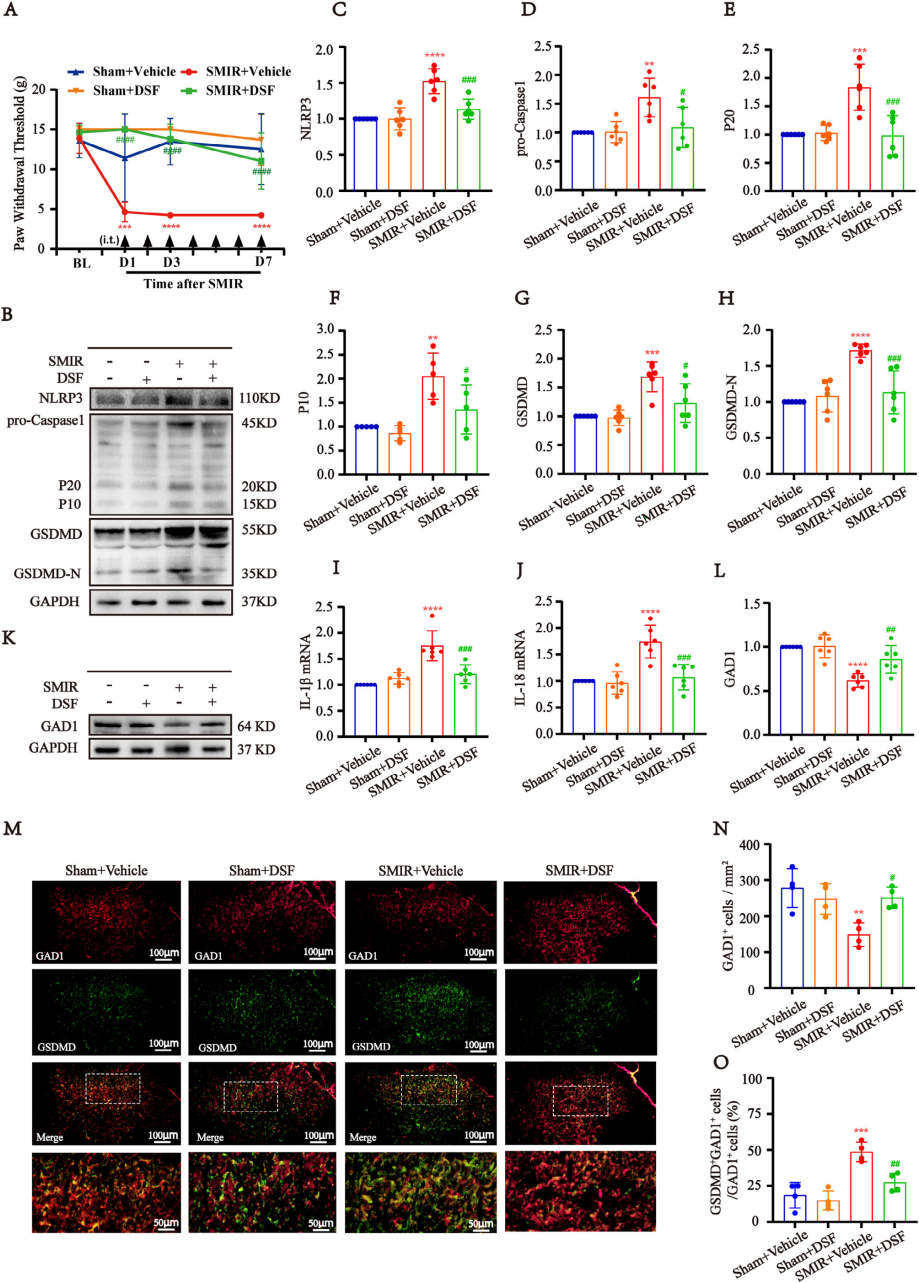
Intrathecal GSDMD inhibitor reversed SMIR‐induced mechanical allodynia through decreasing the pyroptosis of GABAergic neurons in the spinal cord. (A) Repeated injections of DSF (50 μg, i.t.) reverse the SMIR‐induced mechanical allodynia in SMIR rats (*n* = 8). Generalized estimating equations (GEE) model, followed by Bonferroni's post hoc test, ***p < 0.001, ****p < 0.0001 compared with Sham + Vehicle group, ^####^ *p* < 0.0001 compared with SMIR + Vehicle group. (B) Representative western blot images of the NLRP3‐caspase1‐GSDMD pathway expression in vehicle or DSF‐treated SMIR rats. (C–H) Quantification results of western blot showed that the expression of NLRP3, Caspase‐1, P20, P10, GSDMD, and GSDMD‐N were decreased in the SMIR + DSF group compared with the SMIR + Vehicle group (*n* = 6). One‐way ANOVA, followed by Bonferroni's post hoc test, **p < 0.01, ***p < 0.001, ****p < 0.0001 compared with the Sham + Vehicle group, ^##^ *p* < 0.01, ^###^ *p* < 0.001, ^####^ *p* < 0.0001 compared with the SMIR + Vehicle group. (I, J) q‐PCR analysis showed the mRNA expression of the mRNA levels of IL‐1β, IL‐18 were reduced after DSF injection in SMIR rats compared with vehicle (*n* = 6). One‐way ANOVA, followed by Bonferroni's post hoc test, ****p < 0.0001 compared with Sham + Vehicle group, ^###^ *p* < 0.001 compared with SMIR + Vehicle group. (K) Representative western blot images of GAD1 in vehicle or DSF‐treated SMIR rats. (L) Quantification results of western blot showed the expression of GAD1 was significantly increased compared with the SMIR + Vehicle group (*n* = 6). One‐way ANOVA, followed by Bonferroni's post hoc test, ****p < 0.0001 compared with the Sham + Vehicle group, ^##^ *p* < 0.01 compared with the SMIR + Vehicle group. (M) Double immunofluorescence and statistical analysis of GSDMD with GAD1 in vehicle or DSF‐treated SMIR rats. Scale bar: 100 μm and 50 μm. (N) The number of GAD1^+^ cells were evidently reversed at lamina III‐IV of the spinal cord dorsal horn in the SMIR + DSF group compared with the SMIR + Vehicle group (*n* = 4). One‐way ANOVA, **p < 0.01 compared with the Sham + Vehicle group, ^#^ *p* < 0.05 compared with the SMIR + Vehicle group. (O) Intrathecal administration of DSF significantly inhibited the proportion of GSDMD^+^GAD1^+^ GABAergic neurons in SMIR rats compared to vehicle treatment (*n* = 4). One‐way ANOVA, followed by Bonferroni's post hoc test, ***p < 0.001 compared with the Sham + Vehicle group, ^##^ *p* < 0.01 compared with the SMIR + Vehicle group.

Subsequently, DSF (50 μg/d, i.t.) or vehicles (10% DMSO) was administered once daily for seven consecutive days following SMIR or Sham. As shown in Figure 4A, behavioral tests indicated that repeated injection of DSF significantly reversed established mechanical allodynia in SMIR rats. Western blot analysis revealed that the expression of NLRP3, Caspase‐1, P20, P10, GSDMD, and GSDMD‐N were significantly reduced in the SMIR + DSF group compared with the SMIR + Vehicle group (Figure 4B–H). Additionally, the mRNA levels of IL‐1β and IL‐18 were also reduced after DSF injection in SMIR rats compared with vehicle (Figure 4I,J). These results indicated that repeated DSF administration effectively inhibited the NLRP3–Caspase1–GSDMD pathway and reversed SMIR‐induced mechanical allodynia.

Furthermore, we next tested whether the loss of GABAergic neurons was associated with the GSDMD‐mediated pyroptosis. Western blot analysis revealed that, along with the downregulation of the NLRP3‐Caspase‐1‐GSDMD pathway following DSF treatment, the expression of GAD1 was significantly increased compared with the SMIR + Vehicle group (Figure 4K,L). Consistent with these findings, immunofluorescence analysis showed that DSF treatment significantly increased the number of GAD1^+^ cells in the spinal cord and reduced the proportion of GSDMD^+^GAD1^+^ GABAergic neurons in SMIR rats compared with vehicle treatment (Figure 4M–O).

Taken together, these results suggested that intrathecal GSDMD inhibitor reversed SMIR‐induced mechanical allodynia by reducing the pyroptosis of spinal GABAergic neurons.

### 3.4 Spinal Mfn2 Was Downregulated Accompanied by ROS Accumulation after SMIR

**FIGURE 5:**
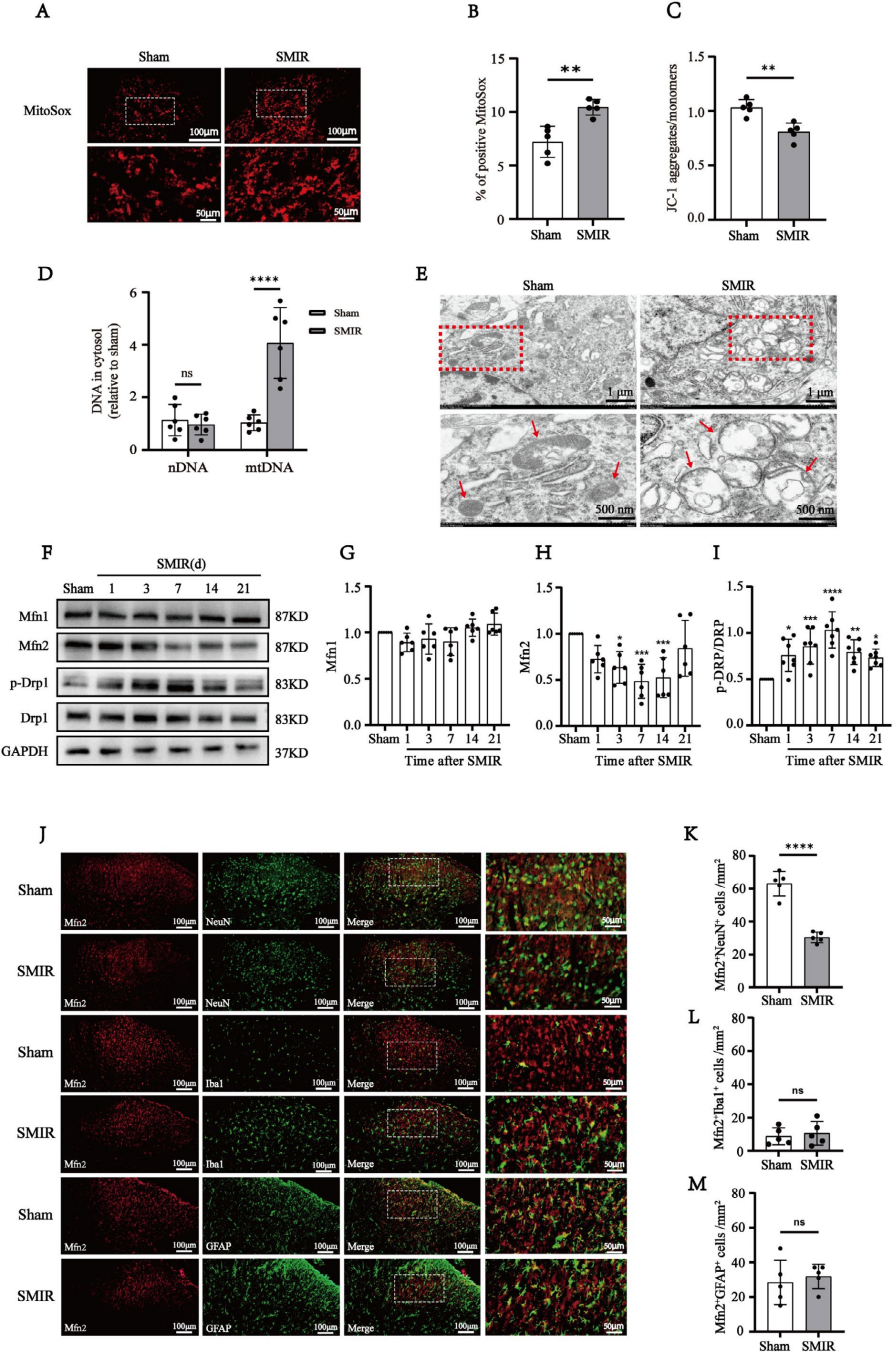
SMIR rats exhibited mitochondrial dysfunction and reduced expression of Mfn2 in the spinal cord. (A, B) The representative images of MitoSox in the spinal of rats in sham and SMIR rats. Scale bar: 100 μm and 50 μm. Quantitative analysis show that the mitochondrial ROS generation was increase in the SMIR rats compared with the Sham group (*n* = 5). Student's *t*‐test, **p < 0.01 compared with the Sham group. (C) JC‐1 staining and quantitative analysis show that MMP was decreased in the SMIR rats compared with the Sham group (*n* = 5). Student's *t*‐test, **p < 0.01 compared with the Sham group. (D) mtDNA level in the cytosol of the SMIR group was higher than that in the Sham group, while there was no significant change in nuclei DNA level (*n* = 6). Student's *t*‐test, ****p < 0.0001 compared with the Sham group. (E) The mitochondrial ultrastructure in spinal neurons exhibited mitochondrial swelling, destruction and disappearance of mitochondrial cristae compared with the Sham group (*n* = 3). The labeled red boxes highlight the mitochondrial ultrastructure. The red arrow represents the mitochondria. Scale bar: 1 μm and 500 nm. (F) Representative Western blot images of the mitochondrial dynamics‐related proteins in the sham or SMIR rats. (G–I) Quantitative analysis showed that the protein levels of Mfn2 was significantly decreased and p‐Drp1 was significantly increased on day 1 and sustained on day 21. While no significant difference was observed in Mfn1 between the sham and SMIR groups (*n* = 6). One‐way ANOVA, followed by Bonferroni's post hoc test, *p < 0.05, **p < 0.01, ***p < 0.001, ****p < 0.0001 compared with the Sham group. (J) Double immunofluorescence of Mfn2 with NeuN, Iba1, GFAP in the spinal cord of the sham and SMIR rats. Scale bar: 100 μm and 50 μm for detail. (K–M) Statistical analysis indicated that the immunoreactivity of Mfn2^+^NeuN^+^ in the SMIR group were significantly decreased compared with the Sham group, while there was no different in the immunoreactivity of Mfn2^+^Iba1^+^ and GSDMD^+^GFAP^+^ between Sham and SMIR groups (*n* = 4). Student's *t*‐test, ****p < 0.0001 compared with the Sham group.

Mitochondria dysfunction is increasingly recognized as a pivotal factor in the peripheral nervous system alterations in both acute inflammatory pain and chronic neuropathic pain [28, 29]. Emerging research suggests that mitochondrial abnormalities can promote excessive reactive oxygen species (ROS) generation, thereby triggering the activation of the NLRP3‐Caspase1‐GSDMD pathway [30, 31]. Therefore, we further explored the molecular mechanisms underlying spinal GABAergic neuron pyroptosis in SMIR rats. In this study, mitochondrial function was assessed by mitochondrial ROS generation, mitochondrial membrane potential (MMP), and cytosolic mitochondrial DNA content using the MitoSox Red reagent, the potentiometric mitochondrial dye JC‐1, and qPCR. We found that the MitoSox immunoreactivity in the spinal dorsal horn was increased (Figure 5A,B), MMP was markedly decreased (Figure 5C) and the spinal cytosolic mitochondrial DNA content was significantly increased (Figure 5D) in the SMIR group compared to that in the Sham group. Furthermore, transmission electron microscopy (TEM) analysis of spinal neurons revealed mitochondrial swelling, structural destruction, and loss of mitochondrial cristae following SMIR (Figure 4E). These findings suggest that impaired mitochondrial function contributes to the activation of GSDMD‐mediated pyroptosis in SMIR rats.

To investigate the mechanisms underlying mitochondrial dysfunction in SMIR rats, we analyzed key proteins involved in mitochondrial dynamics, including the fusion protein Mfn2 and the fission protein dynamin‐related protein 1 (Drp1). Western blot revealed that the protein levels of Mfn2 were significantly decreased and p‐Drp1 was significantly increased on day 1 and sustained on day 21. However, no significant difference was observed in Mfn1 between the Sham and SMIR groups (Figure 5F–I). Given the paucity of studies on Mfn2 in pain, we further performed double immunofluorescence of Mfn2 with neuronal cell‐specific markers, including IBA1 for microglia, GFAP for astrocytes, and NeuN for neurons after 7 days of SMIR to investigate the cellular localization of Mfn2 in the Sham and SMIR groups. In Sham rats, Mfn2 was predominantly colocalized with NeuN and GFAP and to a lesser extent with IBA1 (Figure 5J). However, in SMIR rats, the numbers of Mfn2^+^NeuN^+^ were significantly decreased at day 7 after SMIR, while the numbers of Mfn2^+^GFAP^+^ and Mfn2^+^ Iba1^+^ remained at the basal level (Figure 5K–M). These findings suggest that neuronal Mfn2 downregulation contributes to mitochondrial dysfunction, which is involved in SMIR‐induced mechanical allodynia.

### 3.5 Mfn2 Activator Alleviated SMIR‐Induced Mechanical Allodynia Through Reducing Mitochondrial Dysfunction

To evaluate the effect of Mfn2 on mechanical allodynia following SMIR, we intrathecally injected the Mfn2 activator MASM7 in SMIR rats. As shown in the Figure S1C, MASM7 (2.5, 10, 25 μg, i.t.) or vehicle (10% DMSO) was administered once on day 3 after SMIR to determine the optimal dose. The von Frey test was conducted before MASM7 (2.5 μg) had no effect on the mechanical PWT values of SMIR rats, whereas both the moderate (10 μg) and high (25 μg) doses significantly increased the mechanical PWT values in SMIR rats, which peaked at 1 h and lasted for at least 4 h compared with those in the SMIR + Vehicle group (Figure S1D). These results suggested that a single intrathecal injection of 10 μg or 25 μg MASM7 effectively alleviated SMIR‐induced mechanical allodynia. To minimize the risk of side effects of the higher dose, 10 μg was selected for subsequent experiments in this study.

**FIGURE 6:**
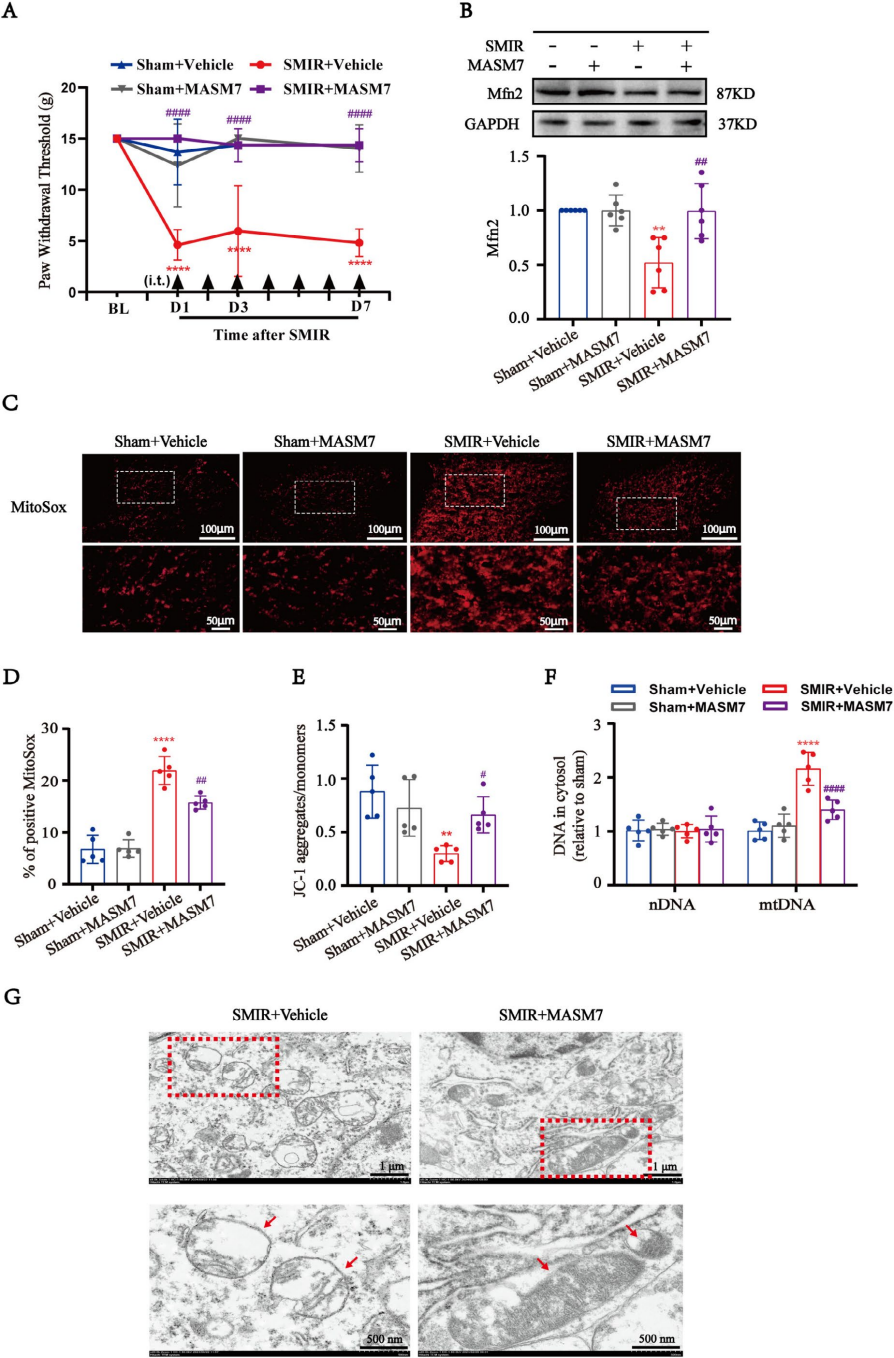
Mfn2 activator alleviated SMIR‐induced mechanical allodynia through reducing mitochondrial dysfunction. (A) Repeated injections of MASM7 (10 μg, i.t.) reversed the established mechanical allodynia in SMIR rats (*n* = 6). Generalized Estimating Equations (GEE) model, followed by Bonferroni's post hoc test, ****p < 0.0001 compared with the Sham + Vehicle group, ^####^ *p* < 0.0001 compared with the SMIR + Vehicle group. (B) Western blot indicated treating with MASM7 increased the expression levels of Mfn2 compared with the SMIR + Vehicle group (*n* = 6). One‐way ANOVA, followed by Bonferroni's post hoc test, **p < 0.01 compared with the Sham + Vehicle group, ^##^ *p* < 0.01 compared with the SMIR + Vehicle group, (C, D) The representative images of MitoSox of rats in vehicle or MASM7‐treated SMIR rats. Scale bar: 100 μm and 50 μm. Quantitative analysis showed that treated with MASM7 significantly decreased the generation of mitochondrial ROS induced by SMIR compared with the Vehicle group (*n* = 5). One‐way ANOVA, followed by Bonferroni's post hoc test, ****p < 0.0001 compared with the Sham + Vehicle group, ^##^ *p* < 0.01 compared with the SMIR + Vehicle group. Scale bar: 100 μm and 50 μm for detail. (E) JC‐1 staining and quantitative analysis show that MMP was revered in the SMIR + MASM7 group compared with the SMIR + Vehicle group (*n* = 5). One‐way ANOVA, followed by Bonferroni's post hoc test, **p < 0.01 compared with the Sham + Vehicle group, ^#^ *p* < 0.05 compared with the SMIR + Vehicle group. (F) The abnormal release of cytosolic mitochondrial DNA in the SMIR + MASM7 group was significantly decreased compared with the SMIR + Vehicle group (*n*=5). One‐way ANOVA, followed by Bonferroni's post hoc test, ****p < 0.0001 compared with the Sham + Vehicle group, ^####^ *p* < 0.0001 compared with the SMIR + Vehicle group. (G) Representative electron morphological changes of the mitochondrial showed the mitochondrial morphological injury in spinal neurons caused by SMIR was alleviated by MASM7 treatment (*n* = 3). The labeled red boxes highlight the mitochondrial ultrastructure. The red arrow represents the mitochondria. Scale bar: 1 μm and 500 nm.

Subsequently, MASM7 (10 μg, i.t.) was administered once daily for 7 consecutive days following SMIR. Mechanical PWT assessments were conducted at 2 h after each daily MASM7 injection. We found that repetitive injections of MASM7 significantly reversed the mechanical allodynia in SMIR rats compared with the SMIR + Vehicle group (Figure 6A).

Following the behavioral assessments, we examined the effects of MASM7 on Mfn2 expression and mitochondrial function in SMIR rats. Western blot analysis showed that the administration of MASM7 significantly increased the expression levels of Mfn2 compared with the SMIR + Vehicle group (Figure 6B). Additionally, compared with the vehicle treatment SMIR group, activation of Mfn2 with MASM7 obviously decreased the MitoSox immunoreactivity, restored the reduction of MMP, and decreased the abnormal release of cytosolic mitochondrial DNA (Figure 5C–F). Furthermore, evaluation of mitochondrial morphology revealed that MASM7 treatment alleviated mitochondrial structural damage in spinal neurons of SMIR rats (Figure 6G).

Therefore, these results substantiated that repeated intrathecal injection of MASM7 alleviated the SMIR‐induced mechanical allodynia by reducing mitochondrial dysfunction in the spinal cord.

### 3.6 Activation of Mfn2 Reduced the GSDMD‐Mediated Pyroptosis of GABAergic Neurons

**FIGURE 7:**
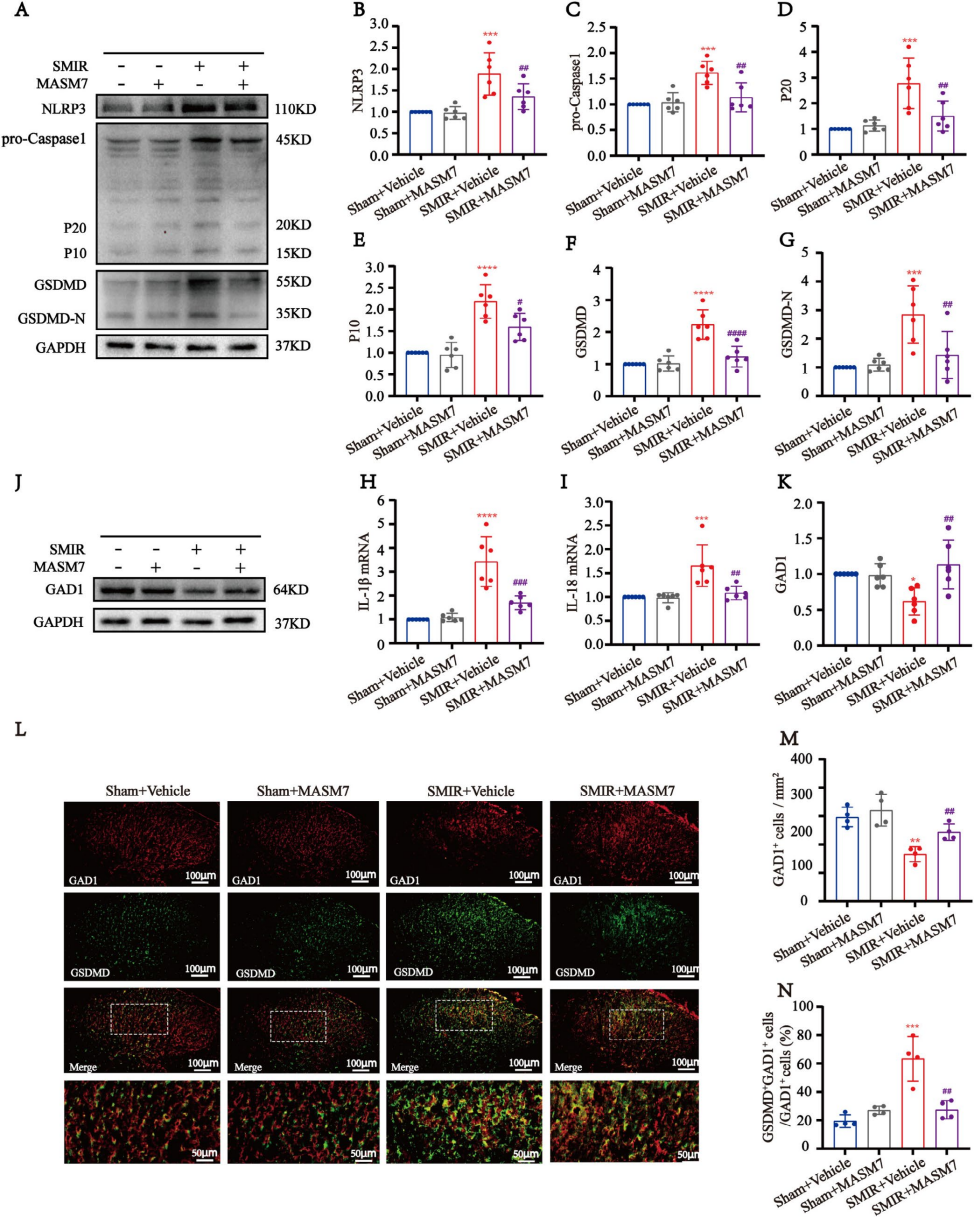
Activation of Mfn2 reduced the GSDMD‐mediated pyroptosis of GABAergic neurons. (A) Representative Western blot images of the NLRP3‐Caspase1‐GSDMD pathway expression in vehicle or MASM7‐treated SMIR rats. (B–G) Quantification results of Western blot showed that the expression of NLRP3, caspase‐1, P20, P10, GSDMD, and GSDMD‐N were decreased in the SMIR + MASM7 group compared with the SMIR + Vehicle group (*n* = 6). One‐way ANOVA, followed by Bonferroni's post hoc test, ***p < 0.001, ****p < 0.0001 compared with the Sham group, ^#^ *p* < 0.05, ^##^ *p* < 0.01, ^####^ *p* < 0.0001 compared with the SMIR + Vehicle group. (H, I) q‐PCR analysis showed the mRNA expression of IL‐1β and IL‐18 were reduced after MASM7 injection in SMIR rats compared with vehicle (*n* = 6). One‐way ANOVA, followed by Bonferroni's post hoc test, ***p < 0.001, ****p < 0.0001 compared with the Sham group, ^##^ *p* < 0.01, ^####^ *p* < 0.0001 compared with the SMIR + Vehicle group. (J) Representative Western blot images of GAD1 in vehicle or MASM7‐treated SMIR rats. (K) Quantification results of Western blot showed the expression of GAD1 was significantly increased in the SMIR + MASM7 group compared with the SMIR + Vehicle group (*n* = 6). One‐way ANOVA, followed by Bonferroni's post hoc test, *p < 0.05 compared with the Sham group, ^##^ *p* < 0.01 compared with the SMIR + Vehicle group. (L) Double immunofluorescence and statistical analysis of GSDMD with GAD1 in vehicle or MASM7‐treated SMIR rats. Scale bar: 100 μm and 50 μm. (M) The number of GAD1^+^ positive cells was evidently reversed at lamina III‐IV of the spinal cord dorsal horn in the SMIR + MASM7 group compared with the SMIR + Vehicle group (*n* = 4). One‐way ANOVA, **p < 0.01 compared with Sham + Vehicle group, ^##^ *p* < 0.01 compared with the SMIR + Vehicle group. (N) Intrathecal administration of MASM7 significantly inhibited the proportion of GSDMD^+^GAD1^+^ GABAergic neurons in SMIR rats as compared to vehicle treatment (*n* = 4). One‐way ANOVA, followed by Bonferroni's post hoc test, ***p < 0.001 compared with the Sham + Vehicle group, ^##^ *p* < 0.01 compared with the SMIR + Vehicle group.

Next, we investigated the effects of MASM7 on the expression of the GSDMD‐mediated pyroptosis in SMIR rats. Western blot analysis revealed that treatment with MASM7 dampened the increased levels of NLRP3, caspase‐1, P20, P10, GSDMD, and GSDMD‐N compared with the SMIR + Vehicle group (Figure 7A–G). Additionally, MASM7 also downregulated the mRNA expressions of IL‐1β and IL‐18 compared with the SMIR + vehicle group (Figure 7H,I). Those findings indicated that the Mfn2 activator MASM7 inhibited the GSDMD‐mediated pyroptosis in SMIR rats.

In addition, western blot revealed that the expression levels of GAD1 were significantly upregulated following MASM7 admission in SMIR rats (Figure 7J,K). Immunofluorescence analysis further confirmed a significant increase in GAD1 expression in the SMIR + MASM7 group compared with the SMIR + Vehicle group (Figure 7L,M). Moreover, MASM7 treatment significantly reduced the colocalization of GAD1 and GSDMD in the spinal cord compared with the SMIR + Vehicle group (Figure 7L–N).

Overall, these results suggested that Mfn2 activation reversed the SMIR‐induced mechanical allodynia, at least in part, by alleviating the pyroptosis of spinal GABAergic neurons.

## 4 Discussion

In this study, we observed that the development of SMIR‐induced mechanical allodynia was associated with the activation of the NLRP3‐Caspase1‐GSDMD pathway and the pyroptosis of GABAergic neurons in the spinal cord. Intrathecal administration of the GSDMD inhibitor (DSF) significantly suppressed the pyroptosis of spinal GABAergic neurons and alleviated SMIR‐induced mechanical allodynia. Furthermore, we found that the expression of Mfn2 was significantly downregulated in SMIR rats, accompanied by mitochondrial dysfunction and ROS accumulation. Intrathecal administration of the Mfn2 activator MASM7 effectively alleviated SMIR‐induced mechanical allodynia by improving mitochondrial function and inhibiting the GSDMD‐mediated spinal GABAergic neuron pyroptosis. Taken together, this study demonstrates that the pyroptosis of spinal GABAergic neurons plays a key role in the development of CPSP. Moreover, activation of Mfn2 mitigates mitochondrial dysfunction and ROS accumulation, thereby reducing the pyroptosis of spinal GABAergic neurons and alleviating SMIR‐induced CPSP.

Pyroptosis is a recently identified form of programmed cell death, characterized by GSDMD‐mediated cellular swelling, loss of membrane integrity, and the release of pro‐inflammatory cytokines [32]. Growing evidence suggests that the activation of pyroptosis‐related proteins plays crucial roles in various neuropathic pain models [33, 34], but their cell‐specific mechanisms are still controversial. Xu et al. [35] found that inhibition of NLRP3 inflammasome‐mediated pyroptosis in microglia reduced neuropathic pain by alleviating neuroinflammation. However, Jia et al. [36] demonstrated that GSDMD and cleaved Caspase‐1 were predominantly colocalized in spinal cord neurons of morphine‐treated rats. This study further supports the central role of neuronal pyroptosis in CPSP; we observed that the expression of GSDMD, a key executor of pyroptosis, was increased in SMIR rats. Immunofluorescence staining further revealed that GSDMD was primarily expressed in neurons. These results indicate that pyrotosis in the spinal cord neurons is involved in the development of CPSP.

Increasing evidence suggests that the reduction of GABAergic inhibitory tone plays a significant role in the development of pain [9, 37, 38, 39]. Decreased GABA levels and reduced GABAergic interneuron activity in the spinal cord have been reported in the neuropathic pain models, which are characterized by reduced GAD1 expression [40, 41]. Moreover, recent studies demonstrated that apoptosis may be the main mechanism mediating the loss of GABAergic neurons [6, 26]. Meanwhile, Ding et al. [25] demonstrated that pharmacologic inhibition of ferroptosis could prevent the loss of GABAergic neurons and alleviate bone cancer pain (BCP) in mice. This study aimed to explore the relationship between pyroptosis and the reduction of GABAergic neurons. We observed that the pain behaviors in SMIR rats were accompanied by a reduction of GAD1 expression. Immunofluorescence staining revealed a higher proportion of GSDMD^+^/GAD1^+^ GABAergic neurons in the SMIR group compared to Sham rats. Further treatment with GSDMD inhibitor DSF upregulated the expression of GAD1 and decreased the proportion of GSDMD^+^/GAD1^+^ GABAergic neurons. These findings suggested that the pyroptosis of spinal GABAergic neurons plays a key role in the development and progression of CPSP.

Mitochondrial dysfunction is a key upstream event in the activation of pyroptosis [42, 43]. Specifically, mitochondrial homeostasis disruption leading to mitochondrial dysfunction, which characterized by decreased MMP, abnormal release of mtDNA, and abnormal accumulation of ROS. The elevated ROS levels, in turn, have been shown to influence pyroptosis at multiple stages, including the priming and activation of the NLRP3 inflammasome and promoting the cleavage of GSDMD [44, 45]. Generation of ROS induces thioredoxin‐interacting protein (TXNIP) dissociation from thioredoxin‐1 (TRX1) in the cytoplasm and promotes its binding to NLRP3, thereby activating the NLRP3 inflammasome [46]. Furthermore, therapeutic interventions targeting mitochondrial dysfunction [47] or ROS [48] have been shown to effectively attenuate neuronal pyroptosis following ischemic stroke. Therefore, maintaining mitochondrial homeostasis plays a key role in inhibiting pyroptosis.

Mfn2, a critical protein mediating the balance of mitochondrial fission and fusion, is essential for reducing ROS accumulation and maintaining mitochondrial homeostasis [22, 49, 50]. Jiang et al. [49] found that limiting mitochondrial recruitment of Mfn2 diminished formation of the mitophagy complex, resulting in accumulation of damaged mitochondria and ROS. Meanwhile, Zhang et al. [51] demonstrated that Mfn2‐mediated extra mitochondrial fission could generate more mitochondrial fragmentation and increased ROS, which promoted tissue damage during acute kidney injury (AKI). However, the role and molecular mechanisms of Mfn2 in chronic pain remain largely unexplored. In our study, we observed that SMIR promoted the levels of mitochondrial fission protein Drp‐1, while the expression of mitochondrial fusion protein Mfn2 was significantly decreased in the neurons of the spinal cord. This imbalance was accompanied by a decrease in MMP, abnormal mtDNA releasing, and ROS accumulation in the spinal cord of SMIR rats, suggesting that the decreased Mfn2 expression was accompanied by accumulation of ROS in CPSP. Administration of the Mfn2 activator MASM7 reduced ROS accumulation and alleviated SMIR‐induced mechanical allodynia. Meanwhile, MASM7 inhibited the expression of the NLRP3‐Caspase1‐GSDMD pathway and GAD1, suggesting that the neuroprotective effect of the Mfn2 activator on CPSP may be related to the suppression of GABAergic neuron pyroptosis. Collectively, these results indicate that Mfn2 downregulation promotes the pyroptosis of spinal GABAergic neurons during the development of chronic pain.

There are still some limitations in the study. First, pharmacological approaches lack cell specificity. Future studies should incorporate transgenic mouse models and viral tools to enhance research validity and precision. Second, we focused only on the pyroptosis in the spinal cord but did not examine other modes of cell death, such as ferroptosis, apoptosis, and necroptosis. Therefore, future studies need to integrate the relationship between multiple modes of cell death and explore the role and therapeutic potential of their common actions in chronic pain. Third, although we observed the pyroptosis of GABAergic neurons in CPSP, the underlying mechanism contributing to chronic pain remains unclear. Further studies should explore how pyroptosis impacts the activity of nociceptive neurons in CPSP.

## 5 Conclusions

In summary, our study demonstrated that downregulation of Mfn2 leads to mitochondrial dysfunction and ROS accumulation, which promotes the pyroptosis of spinal GABAergic neurons during the development of CPSP.

## Author Contributions

**Yingjie Hu:** conceptualization, methodology, investigation, visualization, writing – original draft. **Xiao He:** methodology, investigation, formal analysis, writing – original draft. **Hu Zang:** methodology, investigation, formal analysis. **Yuye Chen:** investigation, formal analysis. **Li Li:** software, data curation. **Tongtong Liu:** formal analysis. **Li Wan:** writing – review and editing. **Chang Zhu:** investigation, resources. **Wenlong Yao:** conceptualization, writing – review and editing, supervision, funding acquisition.

## Consent

The authors have nothing to report.

## Conflicts of Interest

The authors declare no conflicts of interest.

## Supporting information


Figure S1.

Figure S2.

Data S1.

## Data Availability

The data supporting our study's findings are presented in the article. The corresponding author can provide the data upon reasonable request.
