# Supplementary material for: Downregulation of Mfn2 Contributes to Chronic Postsurgical Pain via Inducing the Pyroptosis of GABAergic Neurons in the Spinal Cord
Source: CNS Neurosci Ther. 2025 Jul 9;31(7):e70508. doi: 10.1111/cns.70508 (PMC12238769; doi:10.1111/cns.70508)
Supplement: Supplementary file 1 — Figure S1. [file CNS-31-e70508-s003.docx]

**Supplementary figure 1**


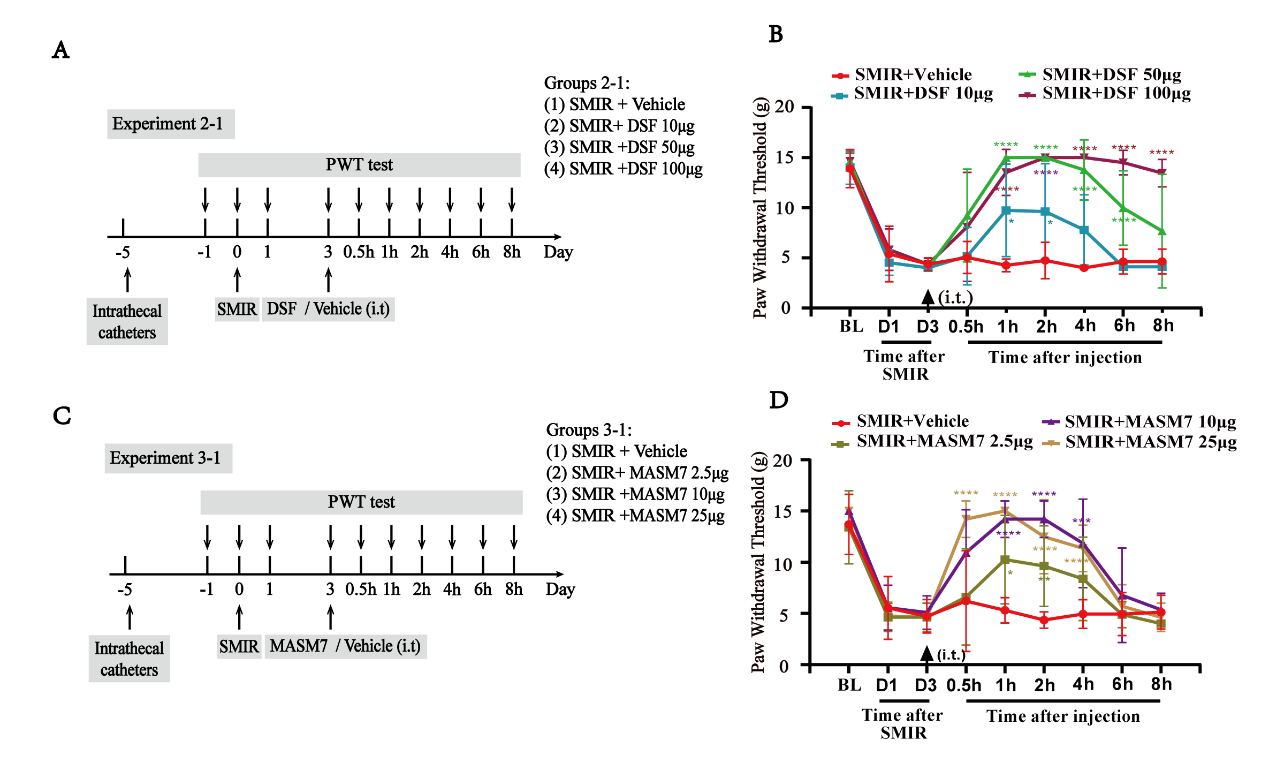


**Supplementary Fig 1. Optimal dose selection of GSDMD inhibitors and Mfn2 activator for the treatment of SMIR. (A-B)** Experimental designs and animal groups for GSDMD inhibitor (DSF, 10, 50, 100μg, intrathecally). PWTs indicated that DSF (50 μg, intrathecally) was the optimal dose for SMIR rats. Generalized Estimating Equations (GEE) model, followed by Bonferroni’s post-hoc test, ^*^*P* < 0.05，^**^*P* < 0.01, ^***^*P* < 0.001, ^****^*P* < 0.0001 compared with the SMIR+Vehicle group. **(C-D)** Experimental designs and animal groups for Mfn2 activator (MASM7, 2.5, 10, 25μg, intrathecally). PWTs indicated that MASM7 (10 μg, intrathecally) was the optimal dose for SMIR rats. Generalized Estimating Equations (GEE) model, followed by Bonferroni’s post-hoc test, ^*^*P* < 0.05，^**^*P* < 0.01, ^***^*P* < 0.001, ^****^*P* < 0.0001 compared with the SMIR+Vehicle group.
