# Supplementary material for: Downregulation of Mfn2 Contributes to Chronic Postsurgical Pain via Inducing the Pyroptosis of GABAergic Neurons in the Spinal Cord
Source: CNS Neurosci Ther. 2025 Jul 9;31(7):e70508. doi: 10.1111/cns.70508 (PMC12238769; doi:10.1111/cns.70508)
Supplement: Supplementary file 2 — Figure S2. [file CNS-31-e70508-s002.docx]

**Supplementary figure 2**


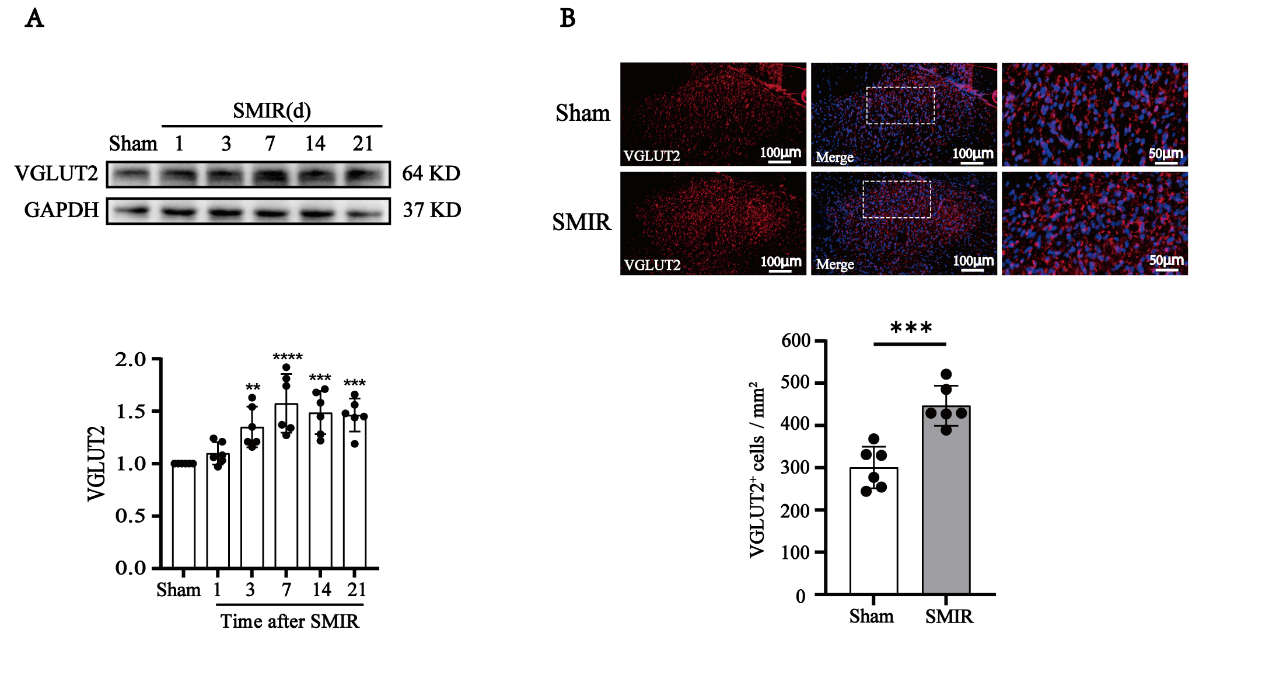


**Supplementary Fig 2. Excitatory neurons were increased in the spinal cord of SMIR rats. (A)** Western blot showed that the level of VGLUT2 in the SMIR rats was significantly increased from days 3 to 21 (n=6). One-way ANOVA, followed by Bonferroni’s post-hoc test, ^**^*P* < 0.01, ^***^*P* < 0.001, ^****^*P* < 0.0001 compared with the Sham group. **(B)** The number of VGLUT2^+^ cells were evidently increased in the spinal cord dorsal horn of SMIR rats at 7 days after surgery (n=5). Student’s t-test, ^****^*P* < 0.0001 compared with the Sham group.
