# Supplementary material for: Downregulation of Mfn2 Contributes to Chronic Postsurgical Pain via Inducing the Pyroptosis of GABAergic Neurons in the Spinal Cord
Source: CNS Neurosci Ther. 2025 Jul 9;31(7):e70508. doi: 10.1111/cns.70508 (PMC12238769; doi:10.1111/cns.70508)

Supplementary file: Full uncropped Blots images

Full unedited blot for Figure 2B

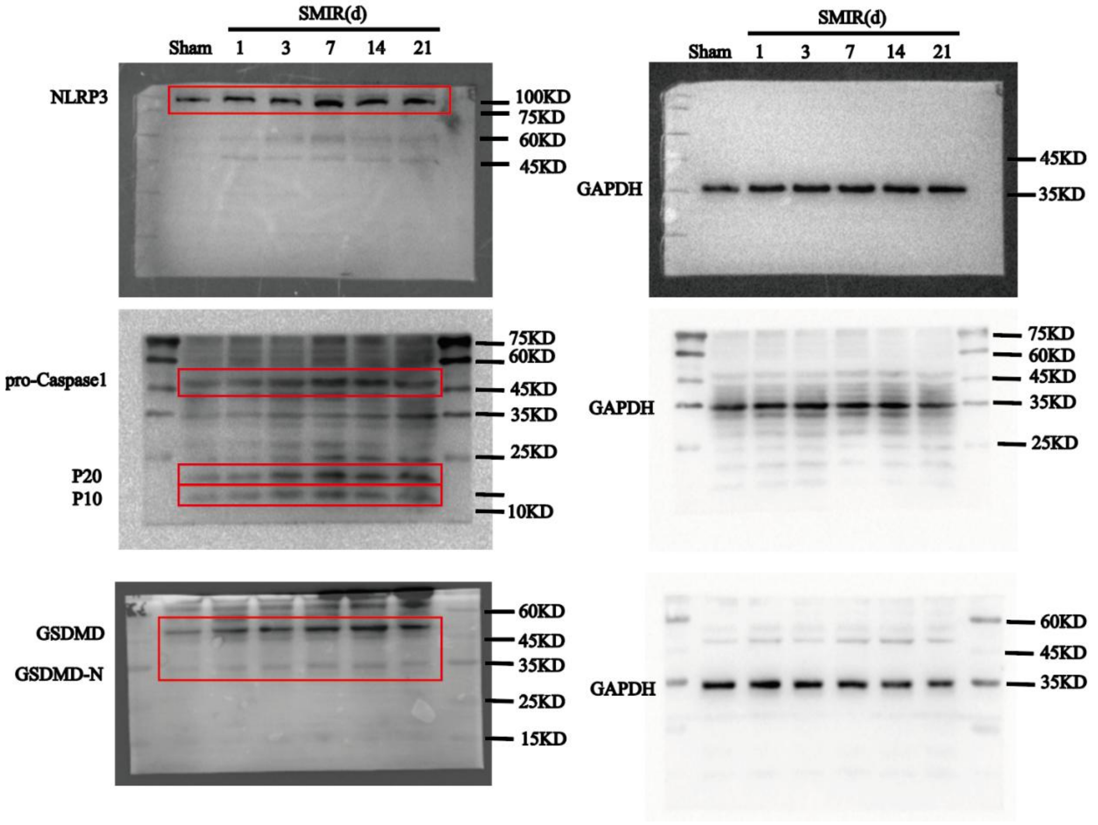

Full unedited blot for Figure 3A

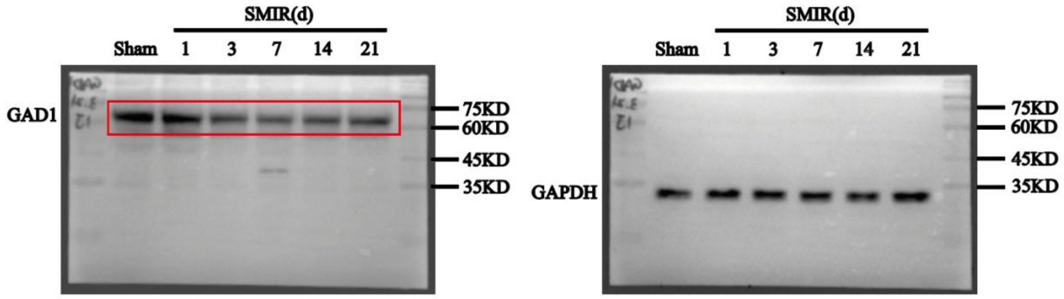

Full unedited blot for Figure 4B

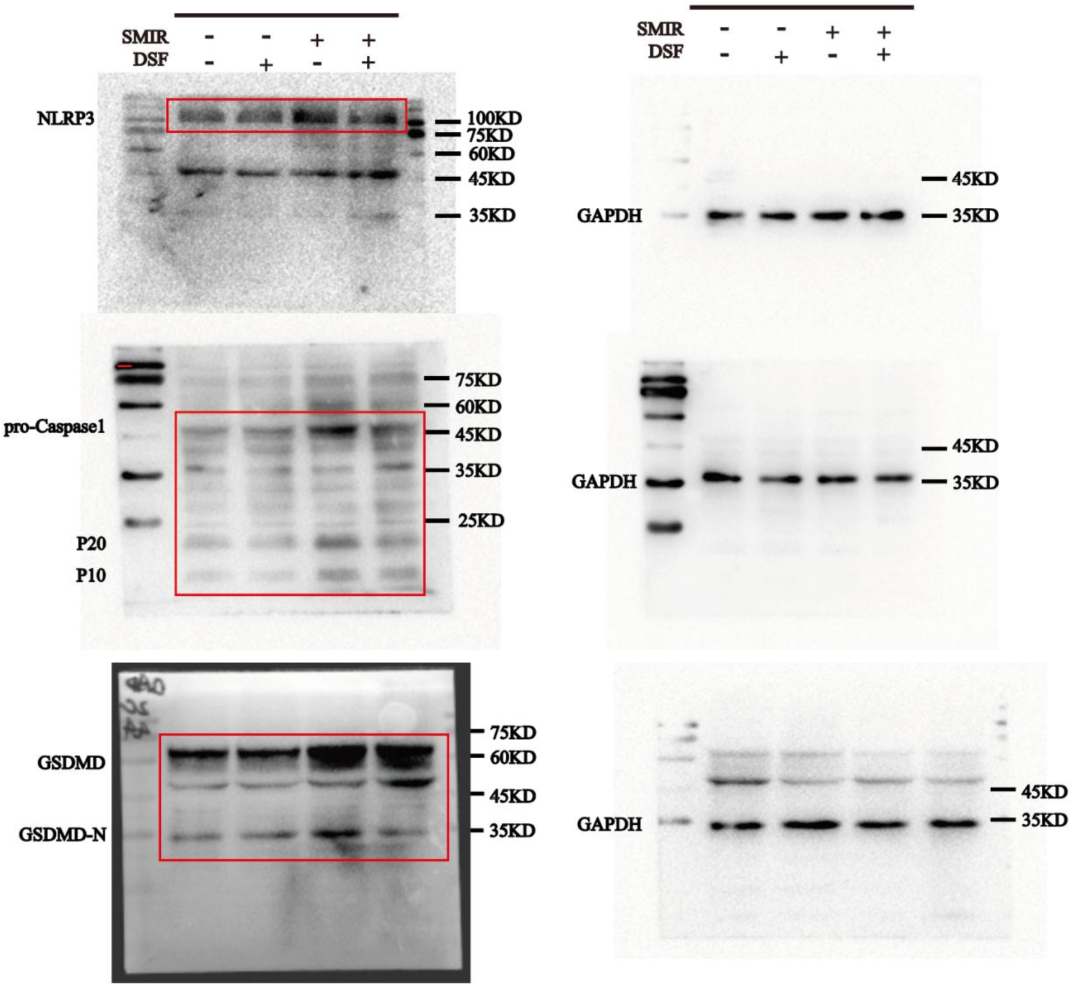

Full unedited blot for Figure 4K

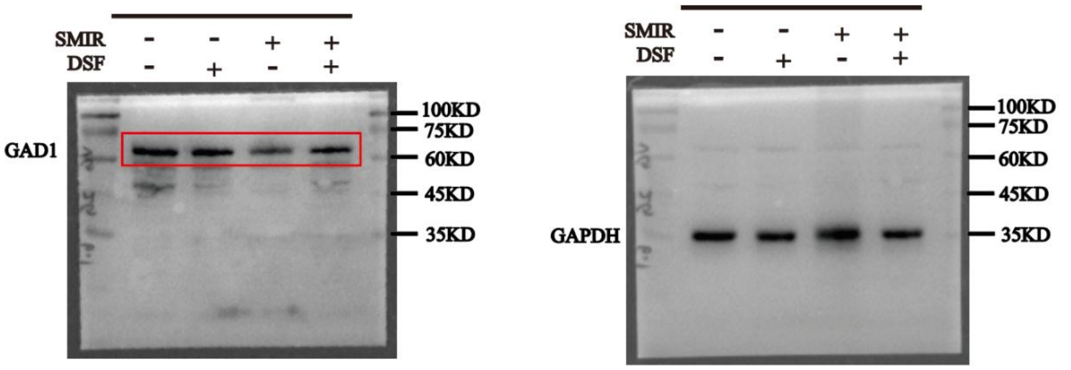

Full unedited blot for Figure 5F

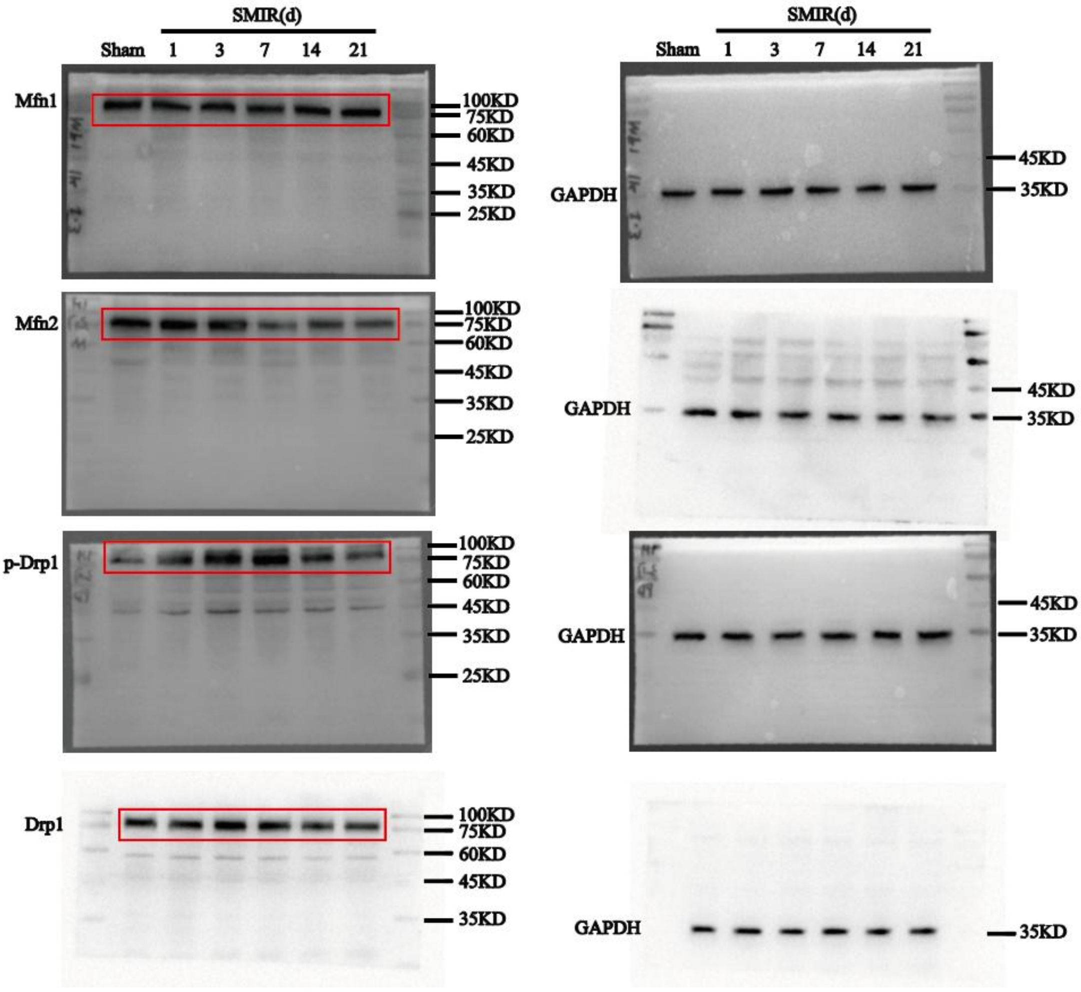

Full unedited blot for Figure 6B

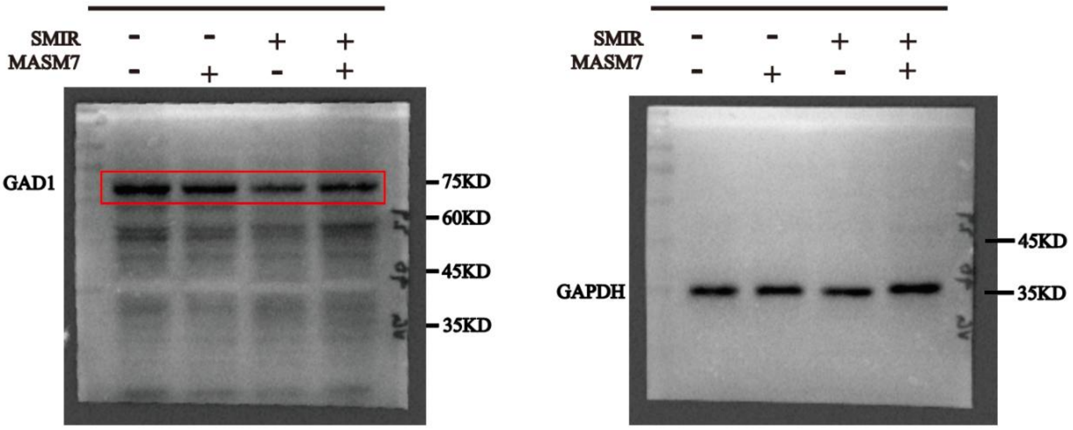

Full unedited blot for Figure 7A

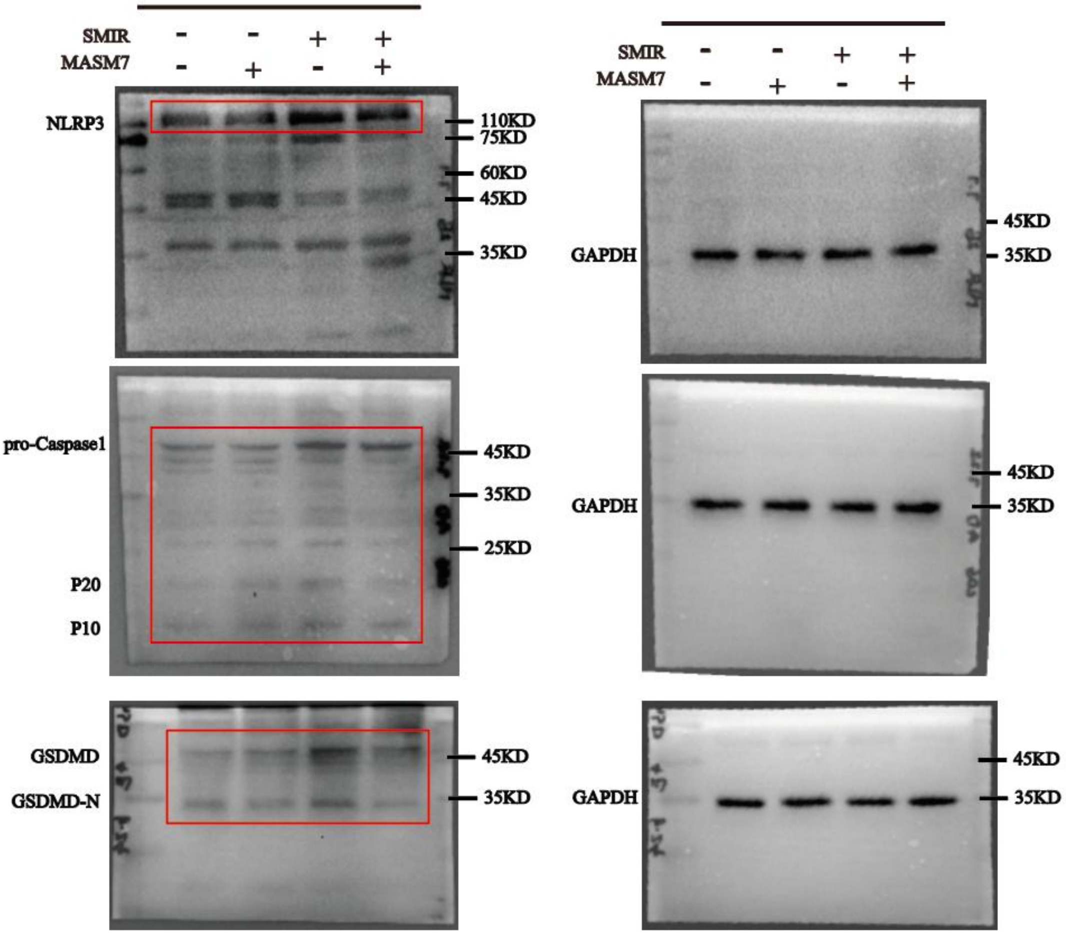

Full unedited blot for Supplementary Fig 2

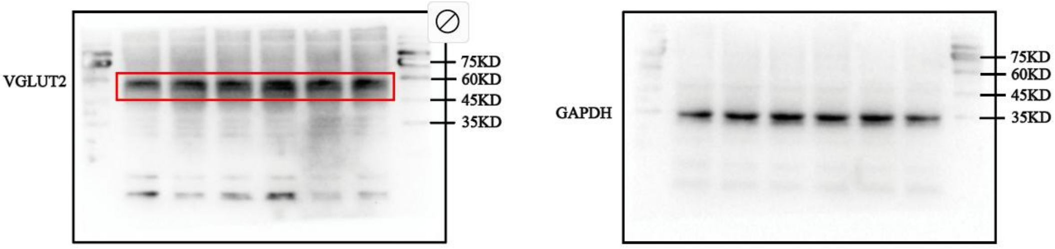

Supplement: Supplementary file 3 — Data S1. [file CNS-31-e70508-s001.pdf]
